# Targeted and Off-Target (Bystander and Abscopal) Effects of Radiation Therapy: Redox Mechanisms and Risk/Benefit Analysis

**DOI:** 10.1089/ars.2017.7267

**Published:** 2018-10-05

**Authors:** Jean-Pierre Pouget, Alexandros G. Georgakilas, Jean-Luc Ravanat

**Affiliations:** ^1^ Institut de Recherche en Cancérologie de Montpellier (IRCM), INSERM, Université de Montpellier, Institut Régional du Cancer de Montpellier (ICM), Montpellier, France.; ^2^ DNA Damage Laboratory, Physics Department, School of Applied Mathematical and Physical Sciences, National Technical University of Athens, Athens, Greece.; ^3^ Univ. Grenoble Alpes, CEA, CNRS INAC SyMMES UMR 5819, Grenoble, France.

**Keywords:** bystander effects, nontargeted effects, abscopal effects, radiotherapy, radionuclide therapy, targeted effects

## Abstract

***Significance:*** Radiation therapy (from external beams to unsealed and sealed radionuclide sources) takes advantage of the detrimental effects of the clustered production of radicals and reactive oxygen species (ROS). Research has mainly focused on the interaction of radiation with water, which is the major constituent of living beings, and with nuclear DNA, which contains the genetic information. This led to the so-called target theory according to which cells have to be hit by ionizing particles to elicit an important biological response, including cell death. In cancer therapy, the Poisson law and linear quadratic mathematical models have been used to describe the probability of hits per cell as a function of the radiation dose.

***Recent Advances:*** However, in the last 20 years, many studies have shown that radiation generates “danger” signals that propagate from irradiated to nonirradiated cells, leading to bystander and other off-target effects.

***Critical Issues:*** Like for targeted effects, redox mechanisms play a key role also in off-target effects through transmission of ROS and reactive nitrogen species (RNS), and also of cytokines, ATP, and extracellular DNA. Particularly, nuclear factor kappa B is essential for triggering self-sustained production of ROS and RNS, thus making the bystander response similar to inflammation. In some therapeutic cases, this phenomenon is associated with recruitment of immune cells that are involved in distant irradiation effects (called “away-from-target” *i.e.*, abscopal effects).

***Future Directions:*** Determining the contribution of targeted and off-target effects in the clinic is still challenging. This has important consequences not only in radiotherapy but also possibly in diagnostic procedures and in radiation protection.

**Table:** 

**Table of Contents**	
I. Introduction	1448
II. Targeted Effects: Oxidative Damage to DNA, Lipids, and Proteins	1448
A. DNA damage, targeted effects	1449
1. Direct effect	1449
2. Indirect effect	1449
B. Effects of irradiation on lipids	1452
C. Protein damage	1453
D. Targeted effects: conclusions	1454
III. Nuclear-Centered View of the Cellular Response to Radiation	1454
A. DNA DSB repair	1454
B. Nuclear-centered view of the cellular response to radiation: conclusion	1456
IV. Off-Target Effects: An Integrated Cell Response to Radiation	1456
A. Bystander effects	1457
1. Intercellular communications between irradiated and nonirradiated cells	1457
2. Reactive oxygen and nitrogen species initiate and propagate bystander effects	1457
a. Mitochondria-dependent ROS production	1459
(1) Mitochondria and ROS endogenous production	1459
(2) Mitochondria and irradiation	1460
b. NAD(P)H oxidase-dependent ROS production	1460
c. ROS and RNS as second messengers	1461
3. Cell membrane response to radiation	1461
a. Cell membrane and lipid rafts	1461
b. Lipid rafts in mitochondria-ER-associated domains	1463
c. Caveolae, a subgroup of membrane lipid rafts	1463
d. Ion channels and lipid rafts	1463
e. The role of Ca^2+^ ions in bystander effects	1463
f. ROS/RNS and growth factor receptor activation	1464
g. MAPKs and the bystander response	1466
4. Central role of NF-κB in the nuclear and extranuclear responses to radiation	1466
a. Nuclear factor kappa B	1466
b. NF-κB and irradiation	1467
5. The COX-2 and iNOS	1468
B. Distant/systemic effects	1468
1. Immune response as the mediator of radiation-induced systemic effects	1468
2. Abscopal effects: historical changes and clinical evidence	1470
C. Off-target effects: an integrated cell response to radiation: conclusion	1471
V. Benefit/Risk Analysis	1471
A. Target theory	1472
B. The clinical relevance of off-target effects might be radiotherapy dependent	1472
C. Bystander effects and radiation protection	1472
D. The biology of low dose and low-dose rate might differ from that of high dose and high-dose rate	1473
E. Off-target effects and radiotherapy efficacy	1473
F. Benefit/risk analysis: conclusion	1474
VI. General Conclusion	1474
VII. Key Points	1475

## I. Introduction

For about one century, the paradigm of radiation biology has been that cells need to be traversed by radiation to be killed. Therefore, in the context of the “targeted effects” of radiation, most research has focused on DNA because it was considered to be the main if not the only target. Radiation-induced DNA single- and double-strand breaks (SSBs and DSBs) and base damage have been identified and quantified. However, other nuclear components, such as lipids and proteins, can also be affected by radiation-targeted effects.

In the 1990s, a shift in the radiation biology paradigm occurred on the basis of the observation that biological effects could be observed also after irradiation of non-nuclear cell compartments (248, 276, 335). Particularly, the existence of dynamic signaling pathways between the various subcellular compartments (nucleus, endoplasmic reticulum [ER], mitochondria, and cell membrane) needs to be taken into account when assessing radiation-induced effects. The rather naive idea that only the nucleus is sensitive to radiation and that the rest of the cell is inert was progressively replaced by a new view that considers intra- and intercellular signaling mechanisms, leading to the expansion of the cell response to radiation in time (long-lasting radiation-induced effects) and also in space (off-target effects, also called nontargeted effects, such as bystander and abscopal effects).

In this review, we first summarize the main targeted and off-target effects and the underlying molecular mechanisms. Then, we discuss the currently available models to predict the therapeutic efficacy and side effects of radiation exposure by taking into account both targeted and off-target effects.

## II. Targeted Effects: Oxidative Damage to DNA, Lipids, and Proteins

The effect of ionizing radiation on cellular constituents has been extensively studied, particularly the formation of DNA lesions because the biological consequences of radiation were mostly attributed to the formation of DNA lesions. In contrast to other biomolecules that are continuously synthetized and decomposed within cells, the cell genome is replicated only once per cell cycle. Therefore, the integrity of the DNA structure is of major importance to maintain the genetic information. For this reason, cells have developed several repair systems to remove DNA lesions and restore DNA integrity. Conversely, altered RNA, protein, and lipid molecules are discarded and replaced by newly synthesized molecules.

### A. DNA damage, targeted effects

**FIG. 1.**
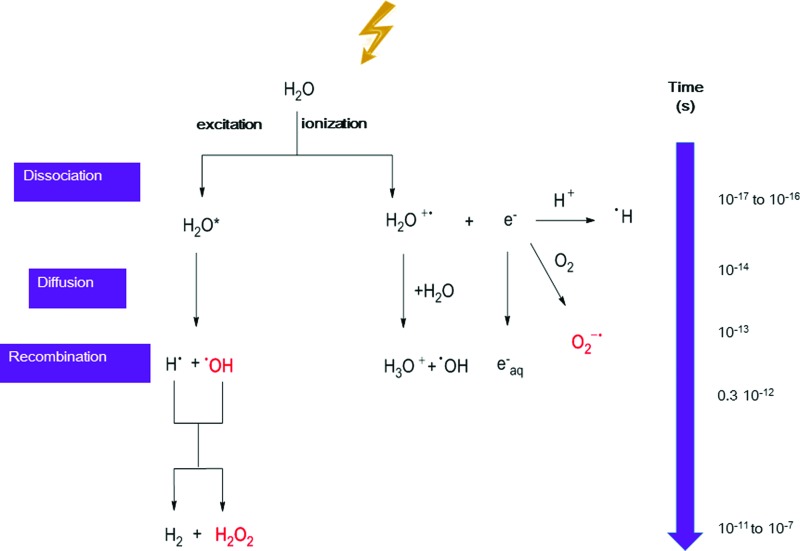
**Kinetic description of the ROS produced by water radiolysis.** Ionizing radiation induces excitation and ionization of water molecules in a very short time. Excited H_2_O* molecules can then dissociate to generate H^•^ and highly reactive HO^•^ that can be produced also by transfer of one proton from ionized water molecules H_2_O^+•^. Ejected electrons can be thermalized to produce hydrated electrons e^−^_aq,_ or react with H^+^ or O_2_ to produce H^•^ and O_2_^−•^ respectively. Radical recombination reactions also can occur, mostly after irradiation with high LET particles, leading to the production, for example, of H_2_O_2_ or H_2_ through recombination of two HO^•^ or H^•^ radicals, respectively. H_2_O_2_, hydrogen peroxide; LET, linear energy transfer; O_2_^−•^, superoxide anion; ROS, reactive oxygen species. To see this illustration in color, the reader is referred to the web version of this article at www.liebertpub.com/ars

Following irradiation, DNA damage can be produced *via* two different mechanisms: (i) direct effect that induces a direct ionization of DNA molecules, and (ii) indirect effect mediated by water radiolysis (214) (Fig. 1). Through this second mechanism, several reactive oxygen species (ROS) can be generated by water radiolysis that can then react with endogenous cellular constituents, including DNA. Most of the DNA damage is attributed to the highly reactive hydroxyl radical HO^•^.

During the last four decades, a considerable amount of work has been done to understand the chemical nature, the mechanism, and the yield of radiation-induced DNA lesions in irradiated cells. Concerning the chemical nature of the DNA modifications, most of the work was performed with isolated nucleosides used as DNA model systems (41). Today, about 80 different DNA modifications (including isomers) have been identified (45). The chemical nature of these modifications is not described in this review article, but information can be found in previous publications (41, 44, 254). Only few examples to highlight the complexity of the undergoing reactions are presented, focusing on lesions that have been observed at the cellular level.

#### 1. Direct effect

Through the direct effect, DNA molecules are directly ionized (loss of an electron), thus generating a DNA radical cation. For each nucleoside, the decomposition of the corresponding radical cation has been described in detail, but the chemistry is different in double-stranded DNA (dsDNA) (46). Indeed, among the DNA constituents, guanine has the lowest oxidation potential. Therefore, even if oxidation occurs on another base or sugar moiety, a fast electron transfer reaction occurs from guanine to the generated radical cation, thus repairing the initially produced radical and generating a guanine radical cation (G^•+^).

**FIG. 2.**
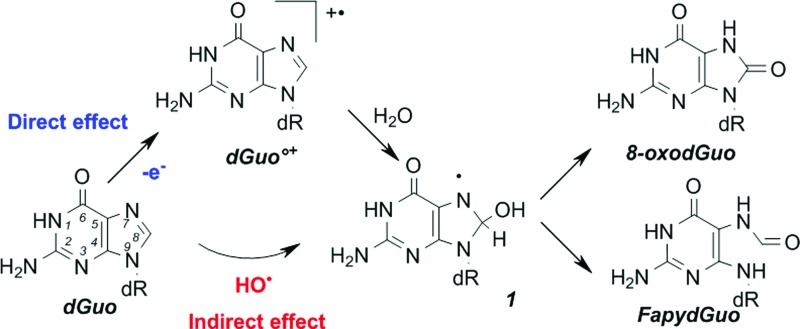
**Mechanisms of 8-oxodGuo and FapydGuo formation through direct or indirect effects of irradiation.** The direct effect produces a guanine radical cation that, following dehydration, produces a neutral radical ***1*** that can also be formed by addition of HO^•^ (produced through the indirect effect) onto the guanine moiety. Oxidation of ***1*** gives rise to 8-oxodGuo, whereas reduction of ***1*** leads to FapydGuo production. 8-oxodGuo, 8-oxo-7′8-dihydro-2′-deoxyguanosine; dGuo, 2′-deoxyguanosine. To see this illustration in color, the reader is referred to the web version of this article at www.liebertpub.com/ars

 Consequently, in dsDNA and in cells, the direct effect of radiation produces mostly unstable guanine radical cations that, after decomposition, give rise typically to two guanine chemical modifications (Fig. 2): 8-oxo-7′8-dihydro-2′-deoxyguanosine (8-oxodGuo) following oxidation and the corresponding formamidopyrimidine derivative FapydGuo on reduction. Interestingly, it has been shown that in irradiated cells, FapydGuo production is two times higher than that of 8-oxodGuo, suggesting that cellular DNA is in a reducing environment.

#### 2. Indirect effect

The radiation-induced DNA lesions produced through the indirect effect are mediated by the initial formation of ROS due to water radiolysis. Exposure of water to ionizing radiation rapidly leads to the generation of HO^•^, ionized water (H_2_O^+^), hydrogen radicals, and hydrated electrons. Then, the reaction of the initially produced radicals generates hydrogen peroxide (H_2_O_2_) and superoxide anion (O_2_^−•^). As these ROS are also produced endogenously and their concentration is regulated by several antioxidant defense mechanisms, the radiation-induced increase of ROS cellular level could damage cellular constituents and also induce oxidative stress (113).

Among the ROS produced upon exposure to radiation, HO^•^ plays a predominant role because it can react, at a diffusion-controlled rate, with almost all biological constituents. Its reactivity within DNA is well documented (43), and about 30% of HO^•^ reacts with the sugar moiety or phosphate group of DNA. Such reaction (69) produces SSBs. HO^•^ reaction with the four different DNA bases has been studied in detail. This reaction produces a plethora of DNA lesions, but is limited by HO^•^ diffusion. Interestingly, the DNA lesions generated by such mechanism are not different from those produced *via* the direct effect, because similar radical intermediates are generated by the two mechanisms. For instance, concerning 8-oxodGuo, addition of HO^•^ to the position C8 of guanine produces radical ***1*** (Fig. 2), also generated following hydration of guanine radical cations arising from direct oxidation of DNA. Similar observations have been made for all DNA bases. One-electron oxidation of DNA bases gives rise to similar chemical modifications as those produced by the indirect effect of irradiation, the only difference concerns their relative yields (42).

The relative importance of the direct and indirect effects in the formation of radiation-induced DNA lesions is still a matter of debate. The general idea is that for low LET (linear energy transfer) radiation such as X- or γ-rays, the direct effect accounts for about 30% of DNA lesions (and thus 70% is attributed to the indirect effect), and that this proportion increases for higher LET radiations such as protons, carbons, and α-particles. A low mean LET of 0.2 keV/μm is observed with γ/X-rays and beta radiation, while LET increases up to 4–26 keV/μm with Auger electrons and up to 50–230 keV/μm with alpha particles. However, according to the chemical mechanism of formation of radiation-induced DNA lesions, the direct effect should mostly produce lesions on the guanine moiety (8-oxodGuo, *e.g.*) as explained above, due to fast electron transfer in DNA. Conversely, the indirect effect should generate lesions in all DNA bases and also strand breaks. Thus, an attempt has been made to determine the relative formation of 8-oxodGuo compared with other oxidative bases in cells after exposure to radiations with different LETs (78). To mimic the direct effect, a laser irradiation at 266 nm was used because it can produce direct DNA ionization following two-photon absorption. As expected, in these conditions, 8-oxodGuo was predominantly formed. This indicates that an efficient electron transfer reaction from guanine to other base radical cations (that are produced by two-photon ionization) takes place also within irradiated cells (*vide supra*). Conversely, the predominance of 8-oxodGuo formation relative to other oxidative DNA lesions in cellular DNA was not observed after gamma or high-LET radiation. Indeed, it has been shown that increasing the particle's LET does not increase the relative formation of 8-oxodGuo compared with other DNA lesions.

This suggests that the direct effect is not increased upon exposure to high LET radiation. Moreover, the strong correlation between HO^•^ radiolytic yield and the number of produced DNA lesions strongly suggests that DNA lesions are predominantly produced *via* the indirect effect (242). Additional work is needed to clarify this point. Particularly, recent studies have highlighted the fact that the guanine radical cation decomposition is affected by its environment. Therefore, the measurement of a specific product generated *via* the direct effect of radiation would be much more informative than measuring 8-oxodGuo formation, because the latter can be produced through the two mechanisms. Indeed, it has been shown that some amino acids chemically repair G^•+^ (192, 193), and that addition of other amino acids as well as polyamines to the C8 position produces other DNA lesions (235, 285, 338), including DNA-protein crosslink. Thus, monitoring lesions specifically produced by the direct effect of radiation could provide additional information to better estimate the direct effect relative contribution.

**FIG. 3.**
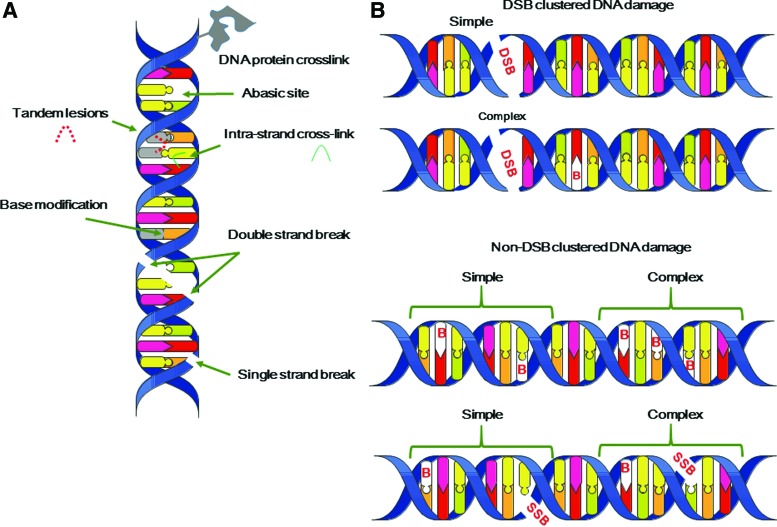
**Radiation-induced DNA damage.** Different types of DNA lesions that could be produced by ionizing radiations, including **(A)** base **(B)** modifications, abasic sites, SSB or DSB, intrastrand crosslink, tandem DNA lesions involving two adjacent modifications. **(B)** Types of DSB and non-DSB clustered DNA lesions involving the combination of all possible DNA lesions in one or two DNA helix turns (82). DSB, double-strand break; SSB, single-strand break. To see this illustration in color, the reader is referred to the web version of this article at www.liebertpub.com/ars

Finally, it is interesting to note that the chemical nature of radiation-induced DNA lesions is not different from that of DNA lesions produced in cells subject to endogenous oxidative stress (45). This is not surprising because ROS produced by radiation through water radiolysis are similar to those produced endogenously, for example, HO^•^ through the Fenton reaction (204). However, the major difference concerns the lesion three-dimensional (3D) localization (212, 215, 330). Endogenous oxidative stress produces oxidative lesions that should be randomly distributed on the DNA macromolecules. These lesions, including modified DNA bases and SSBs, can be rapidly and efficiently repaired by the cell machinery, simply by removing the modification and using the complementary strand to resynthesize the original DNA sequence. After exposure to ionizing radiations, ROS are locally produced along the particle track and this could generate several DNA modifications at the same site (called “multiple damage sites,” MDS, or clustered DNA lesions) (Fig. 3). These lesions are defined as two or more modifications per helix turn (172, 219). DNA DSBs are one of the best examples of MDS. DSBs are produced in cells even after exposure to very low doses of radiations, and their formation yield increases almost linearly with the dose (102, 260, 329).

In addition, high-LET radiations, which induce more dense ionizations, produce, relatively to SSBs, more DSBs compared with low-LET radiations. In fact, when the LET of the radiation increases, the number of DNA lesions per unit dose (Gy) decreases (242) (due to an increased probability of radical recombination), but at the same time, MDS complexity increases (*i.e.*, the MDS includes more DNA damage types, such as base modifications and strand breaks). MDS repair by the cell machinery is much more difficult as soon as the two DNA strands are modified (262). Thus, this explains why the harmful effect of radiation increases with higher LET, and also why the effect of radiation is mostly attributed to cluster DNA lesions (including DSBs and non-DSB cluster lesions) produced by several ionizations.

**FIG. 4.**
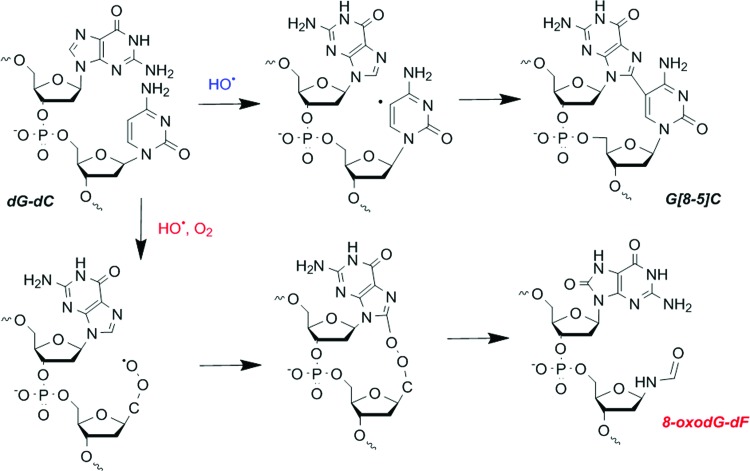
**Mechanisms of formation of complex DNA lesions (including tandem 8-oxodGuo-dF lesions and G[8-5]C intrastrand crosslinks) at dG-dC sequences mediated by a single oxidation event.** After HO^•^ reaction on the cytosine base, in the absence of oxygen, the produced radical can react with an adjacent guanine base, thus producing a G[8-6]C intrastrand crosslink. On the contrary, in the presence of oxygen, the cytosine radical is trapped by molecular oxygen, thus producing a peroxyl radical. This peroxyl radical can react with an adjacent purine base, thus generating an unstable endoperoxide that, on decomposition, gives rise to tandem lesions constituted of two adjacent oxidative DNA lesions. To see this illustration in color, the reader is referred to the web version of this article at www.liebertpub.com/ars

However, recent works have highlighted the fact that a single oxidation event also can produce several modifications (called tandem lesions), composed of two adjacent modifications. For example, as illustrated in Figure 4 for a dG-dC sequence, in the absence of oxygen, crosslinks between two adjacent DNA bases could be produced, generating intrastrand crosslinks (G[8-5]C adduct) (128). Within the same sequence, in the presence of oxygen, the reaction of the initially produced radical with O_2_ produces a hydroperoxide radical that can add to the C8 position of an adjacent guanine (or adenine) base. The resulting unstable endoperoxide undergoes decomposition, producing tandem DNA lesions that involve two adjacent DNA modifications (253), such as 8-oxodGuo and a formylamine residue (8-oxodGuo-dF, Fig. 4) (29). The latter observation, although obtained using isolated DNA, indicates that a radical produced on a DNA base can react with surrounding molecules and particularly with adjacent bases, giving rise to possibly two adjacent modifications.

Moreover, it has been shown that the repair of 8-oxodGuo involved in tandem lesions is significantly reduced compared with the repair of a single lesion (19). At the cellular level, the decomposition reactions of an initially produced radical will strongly depend on its environment. The fact that carbon-centered radicals can react efficiently with molecular oxygen provides a partial explanation for the well-known oxygen enhancement effect, observed in radiation biology. As illustrated in Figure 4, the DNA lesions generated in the absence of oxygen could be different from those created in oxic conditions. Low oxygen concentration could indirectly increase the lifetime of the initially produced radicals, thus favoring the possibility of radical recombination and ultimately decreasing the amount of radiation-induced DNA lesions (250). In addition, the increased lifetime of radicals could also promote repair, for example, by electron donation from mild reducing amino acids (193) or antioxidant molecules that could be used as radioprotectors (192).

This could provide a possible explanation for the reduced level of lesions produced under hypoxic or anoxic conditions (154). Nevertheless, increasing the lifetime of a radical could also favor its reaction with other cellular constituents, leading to the generation of more complex DNA lesions that could compromise DNA repair (154). Indeed, the presence of polyamines, which are highly concentrated in the nucleus of eukaryotic cells, or of a nucleophilic amino acid could lead, respectively, to the formation of DNA adducts (285) and DNA-protein crosslinks (206). These DNA damage types are repaired less efficiently than single lesions (19). These examples illustrate the complexity of the chemical reactions that can occur in cellular DNA following exposure of cells to ionizing radiation (47) and that can vary in oxic and hypoxic conditions.

Although the literature on damage to mitochondrial DNA (mtDNA) is less abundant, mtDNA also is affected by irradiation (358), and additional work is needed to better understand the consequences of this damage (143). This is also true for the nucleotide pool (110) and RNA that are more sensitive to oxidative stress (126) than DNA, which is compacted and protected within the nucleus.

### B. Effects of irradiation on lipids

**FIG. 5.**
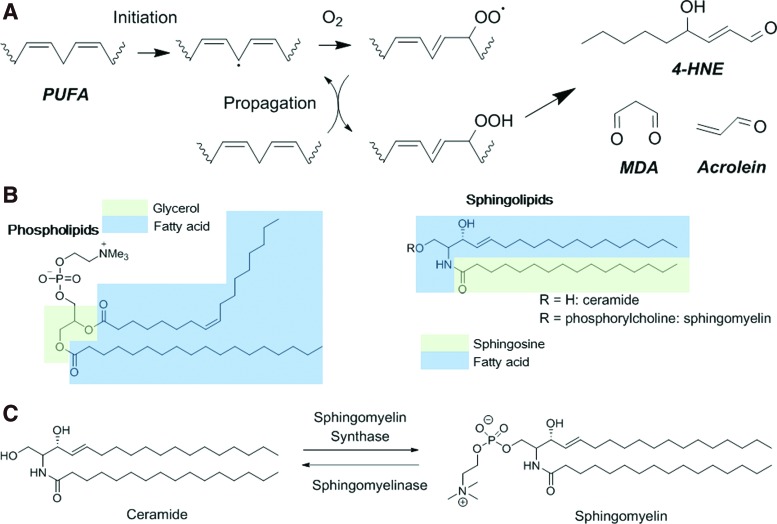
**Radiation-induced lipid peroxidation and sphingolipids.** **(A)** Lipid peroxidation and formation of PUFA decomposition products. **(B)** Phospholipids contain two hydrophobic long-chain fatty acids linked to an alcohol (usually glycerol) and a hydrophilic group made of a phosphate group. Similarly, sphingolipids contain a long-chain sphingoid base (such as sphingosine) linked *via* an amide to long-chain fatty acids, and to one polar head group that makes them amphipathic molecules. Head groups differentiate sphingolipids from ceramides (phosphorylcholine constituting SM and hydroxyl group, respectively). **(C)** Irradiation induces rapid formation of ceramide through the hydrolysis of SM by ASMase. 4-HNE, 4-hydroxy-2-nonenal; ASMase, acid sphingomyelinase; MDA, malondialdehyde; PUFA, polyunsaturated fatty acids; SM, sphingomyelin. To see this illustration in color, the reader is referred to the web version of this article at www.liebertpub.com/ars

The lipid layer of cell membranes is also a radiation target (59, 296). Damage to lipids is mediated by ROS reaction with polyunsaturated fatty acids (PUFA) (209). The initial step involves the abstraction of the bisallylic position of PUFA (Fig. 5A) either by HO^•^ or mediated by a thiyl radical (RS^•^). The produced carbon-centered radical is converted into a peroxyl radical after reaction with molecular oxygen at a diffusion-controlled rate. Then, the peroxyl radical can abstract a hydrogen atom from another PUFA, inducing the so-called peroxidation reactions (271). Decomposition of the hydroperoxide generates breakdown molecules that contain reactive carbonyl groups, such as malondialdehyde (MDA), acrolein, and 4-hydroxy-2-nonenal (4-HNE). These decomposition products could be used as markers of lipid peroxidation reactions. Moreover, if they are not trapped by the cellular defenses (particularly glutathione [GSH]), they could generate secondary decomposition products because they can react with cellular biomolecules (*e.g.*, DNA, RNA, and also amino acids) to generate adducts.

Lipid peroxidation can cause various biological effects: increase in membrane permeability, disruption of ion gradients and transmembrane processes, as well as alteration of the activity of membrane-associated proteins. Besides peroxidation reactions, irradiation could also induce *cis* to *trans* isomerization of PUFA (191), thus affecting the cellular membrane fluidity (194).

It has been clearly shown that the sphingolipid metabolism is altered following exposure to ionizing radiations. Sphingolipids are one of the four major lipid constituents of the cell membrane, in addition to phospholipids, sterols, and glycolipids. Irradiation induces rapid formation of ceramide through the hydrolysis of sphingolipid hydrolases and the concomitant decrease of sphingomyelin at the plasma membrane. Ceramide could be considered a second messenger that not only has an effect on the plasma membrane but also on intracellular signaling molecules (59).

### C. Protein damage

An indirect evidence that ionizing radiations do not target only DNA but also highly abundant proteins is given by the effects of irradiation directed to the cytoplasm (353). The effects observed for doses as low as 1 Gy are changes in protein expression and activity that are mostly mediated by post-transcriptional modifications (302) and also by chemical modifications, including oxidation or carbonylation (255, 295).

Like for DNA, a considerable amount of work has been done to determine the reactivity of amino acids with radicals produced by irradiation (295). Due to its fast and high reactivity, HO^•^ can react with all amino acids with a reaction rate between 10^7^ and 10^10^ *M*^−1^s^−1^. With proteins, the reaction depends mostly on HO^•^ accessibility to amino acids, used for hydroxyl radical footprinting by mass spectrometry to detect structural changes in protein conformation (130). The main reaction pathway involves hydrogen attraction to produce α-carbonyl radicals. Such radicals react with molecular oxygen at a diffusion-controlled rate to produce the corresponding peroxyl radicals that further decompose, inducing fragmentation of the protein backbone. Hydrogen atom abstraction could also occur on the aliphatic chain of amino acids. Similar to what observed for lipids, following the reaction with molecular oxygen, the generated protein hydroperoxides could also induce chain reactions in proteins, leading to a greater loss of amino acids than for the initially formed radical (65). HO^•^ reaction with aromatic amino acids occurs by addition onto the aromatic ring. Addition onto tyrosine (Tyr) residues produces the phenoxyl radical that could induce the formation of tyrosine dimers, involved in the formation of intra- or interprotein crosslinks. Cysteine residues have the lowest redox potential among amino acids; therefore, oxidation of their thiol groups is the major modification. Two-electron oxidation of these residues generates sulfenic acid as the initial cysteine oxidation product that may be converted to disulfides by GSH. Disulfides could be converted back to thiols by disulfide reductases. If not trapped by endogenous thiols, sulfenic acid can be oxidized to generate sulfinic and sulfonic acid.

In addition to oxidation, proteins could also be modified by reduction, through reductant species produced by irradiation (*i.e.*, e_aq_^−^ and H^•^). These reductant species react mostly with thiol groups, and the reaction of H^•^ with methionine generates a sulfuranyl radical that decomposes to produce a carbon-centered radical. The latter is the precursor of homoserine. H^•^ could also abstract a hydrogen atom and reduce cysteine to produce a thiyl radical. Methionine reaction with e_aq_^−^ induces a deamination reaction that causes a peptide break. In addition, e_aq_^−^ reaction with cysteine generates hydrosulfide ions and an alkyl radical, whereas reaction with disulfides produces a thiyl radical and a thiolate ion. Reactive aldehydes, derived from lipid peroxidation, also can react with proteins, mostly with the nucleophilic moieties of amino acids in proteins (209) or DNA bases (25) to generate adducts and crosslinks.

### D. Targeted effects: conclusions

It is now very well documented that irradiation damages cellular biomolecules, such as DNA, RNA, proteins, and lipids. Most of the decomposition reactions of biomolecules mediated by ROS produced during irradiation have been identified. However, the complexity of the cellular media and the possible reaction of transiently produced radicals with the surrounding molecules lead to a great variety of cellular processes induced by radiation that makes almost impossible their precise description/identification. This is a matter of concern for DNA lesions and it cannot be totally excluded that not yet identified lesions are produced in cells exposed to ionizing radiation. The most harmful lesions are not necessarily those produced in higher yields, such as base lesions or SSBs that can be repaired rapidly and with high fidelity. Radiation-induced lesions are not different from those produced by endogenous oxidative stress. However, due to their special distribution in clusters, which hampers their repair, they can be very harmful for the cells.

## III. Nuclear-Centered View of the Cellular Response to Radiation

While SSBs and base damages are efficiently repaired by SSB repair (SSBR), base excision repair (BER), and nucleotide excision repair (NER), DNA DSBs and clustered DNA lesions in general are mainly responsible for the final cellular outcome on irradiation (269).

### A. DNA DSB repair

**FIG. 6.**
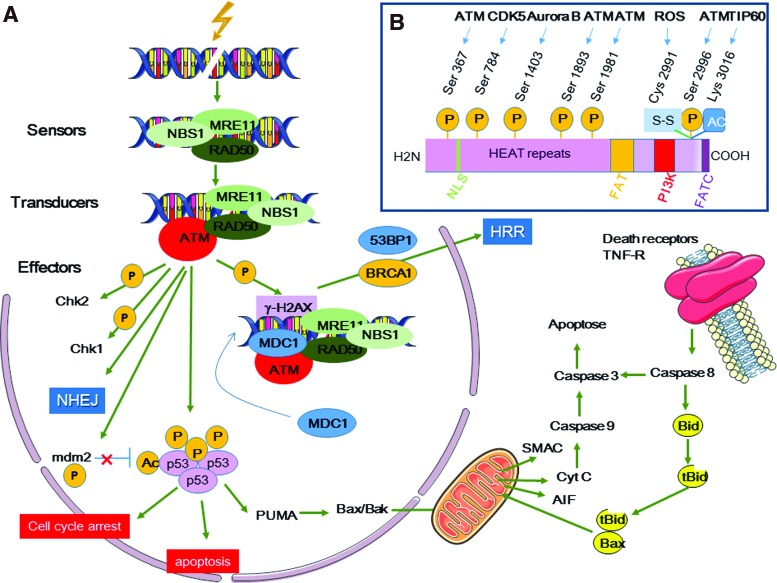
**DNA damage-mediated activation of ATM signaling pathways. (A)** The DNA damage repair mechanism includes sensors, transducers, and effectors. Sensors, such as the MRN complex, recognize the DNA structure modifications induced by DNA DSBs. Transducers are ATM (and ATR involved in HRR) PI3K involved in phosphorylation of many effector proteins that control cell cycle progression (CHK1 and CHK2), DNA repair (*via*, for instance, NHEJ mechanisms), and apoptosis. The intrinsic apoptotic signaling pathway relies on p53 stabilization, followed by PUMA transcription and BAX activation. MDM2 ubiquitin ligase activity plays a central role in keeping a low p53 concentration in non-ATM-activated cells. On ATM activation, MDM2 ubiquitin ligase activity is inhibited and p53 stabilized. PUMA frees the proapoptotic BAX/BAK proteins (members of the BCL2-family) that can transfer to mitochondria where they promote cytochrome C, AIF, and SMAC release and further activation of caspases. In the extrinsic apoptotic signaling pathway, death receptor (TNF-R, TRAIL-R, and FAS) activation leads to BID cleavage by caspase 8 and further release of cytochrome C. ATM-mediated phosphorylation of H2AX triggers recruitment of MDC1, which in turn recruits more MRN complexes and activated ATM to the damaged chromatin. This promotes H2AX phosphorylation and spreads ATM and H2AX phosphorylation over a large chromatin domain. **(B)** ATM structure. ATM is a 350 kDa protein (3056 amino acids) and a member of the PIKK family. It includes HEAT repeats, FAT, PI3K, and FATC domains. The HEAT repeats allow NBS1 binding. Post-translational modifications include autophosphorylation (at Ser 1981, Ser 367, Ser 1893, Ser 2996) and TIP 60 acetylation at Lys 3016 (170, 185, 231, 282). 53BP1, p53-binding protein 1; AIF, apoptosis-inducing factor; ATM, ataxia-telangiectasia mutated; ATR, ATM- and Rad3-related; BID, BH3 interacting-domain death agonist; BRCA1, breast cancer type 1; CHK1, checkpoint kinase 1; CHK2, checkpoint kinase 2; FAT, FRAP-ATM-TRRAP; FATC, FAT C-terminal; HEAT, Huntington-elongation factor 3-protein phosphatase 2A-TOR1; HRR, homologous recombination repair; MDC1, mediator of DNA damage checkpoint protein 1; MDM2, mouse double minute 2 homologue; MRN, MRE11–RAD50–NBS1; NHEJ, nonhomologous end joining; PI3K, phosphatidylinositol-3-kinase; PIKK, phosphatidylinositol 3-kinase-related kinase; PUMA, p53-upregulated modulator of apoptosis; SMAC, second mitochondria-derived activator of caspases; TNF, tumor necrosis factor; TRAIL, TNF-related apoptosis-inducing ligand. To see this illustration in color, the reader is referred to the web version of this article at www.liebertpub.com/ars

DNA DSBs are detected by surveillance proteins of the phosphatidylinositol-3-kinase (PI3K)-like kinase family, particularly the serine/threonine protein kinase ataxia-telangiectasia mutated (ATM), which is the main sensor of DNA damage, and ATM- and Rad3-related (ATR) protein (Fig. 6) (185, 231, 282). Both are actively recruited to DNA DSB sites to monitor the DNA damage response (DDR).

DDR is activated to avoid the transmission of erroneous genetic information to daughter cells and to protect cells from deregulated metabolism. It includes mechanisms involved in DNA damage detection, signaling, and repair, when possible (135), or in the initiation of programmed cell death or senescence.

ATM activation is mediated *via* autophosphorylation on serine 1981 (a hallmark of activated human ATM); however, three additional autophosphorylation sites have been identified as well as a TIP60-mediated acetylation site [for review (282)] (Fig. 6). ATM activation leads to dissociation of ATM homodimers into monomers that will phosphorylate and activate downstream protein kinases. These molecules will act as transducers and effectors and in turn phosphorylate their own substrate(s). One of the first identified targets of ATM phosphorylation was p53 that plays a central role in the radiation response. In physiological conditions, p53 has a short half-life and is maintained at low levels by continuous ubiquitination catalyzed by mouse double minute 2 homologue (MDM2). Upon phosphorylation, p53 is stabilized and accumulates and can activate proteins such as p21 involved in cell cycle arrest by acting on cyclin-dependent kinases (CDK). G1-S, intra S, and G2-M arrest give time for the cells to repair lesions before entering mitosis. Moreover, p53 participates in the activation of the intrinsic apoptosis pathway, for instance, by activating proapoptotic molecules of the BCL2 family, such as p53 upregulated modulator of apoptosis (PUMA) that is involved in the release of proapoptotic factors (BAX/BAK) (Fig. 6). Therefore, p53 activity can drive the final outcome of irradiated cells toward DNA repair or apoptosis *via* a mitochondria-mediated cell death process.

However, ATM full activation requires the recruitment of the MRE11–RAD50–NBS1 (MRN) complex at DNA DSB sites before induction of the DDR systems (nonhomologous end-joining [NHEJ], and homology-directed repair, HDR). The MRN complex also is phosphorylated by ATM. This complex forms a bridge between DNA ends. Then, its nuclease activity, mediated by the nuclease MRE11, resects DNA DSB ends, a crucial step for homologous recombination repair (HRR) (282). Moreover, interaction between NBS1 and ATM is essential for maintaining ATM at DSB sites. Breast cancer type 1 (BRCA1) and p53-binding protein 1 (53BP1) are involved in this interaction. ATM also interacts with a mediator of DNA damage checkpoint protein 1 (MDC1) (170, 185, 231, 282). MDC1 is a DNA damage sensor protein located at DSB sites where it binds to phosphorylated histone H2AX (γH2AX) and ATM. Additional H2AX phosphorylation by ATM allows the recruitment of more MDC1 molecules that bind to ATM and γH2AX in a positive feedback loop, leading to ATM and γH2AX spread over large domains (>500 kb) around DNA breaks and amplification of DNA DSB signaling (136, 170, 185, 231, 282).

Other ATM substrates are proteins involved in G1-S (p21), intra-S (Fanconi anemia group D2 protein [FANCD2], BRCA1, and structural maintenance of chromosomes protein 1 [SMC1]), and G2-M cell cycle arrest (checkpoint kinase 2 [CHK2] or DNA repair [poly(ADP-ribose) polymerase 1 (PARP1), the nuclease Artemis, C-terminal-binding protein 1-interacting protein (CtIP), DNA-dependent protein kinase (DNA-PK) cs84, polynucleotide kinase 3′-phosphatase, DNA-PK, and AKT]. These factors allow DNA repair according to the NHEJ pathway before replication and mitosis (147, 163). ATM is also activated and dissociated into monomers by ROS *via* TIP60 acetylation near the FRAP-ATM-TRRAP C terminal (FATC) domains. Moreover, H2AX phosphorylation, sumoylation, and ubiquitination at DSB sites participate in the recruitment of BRCA1 and 53BP1, two proteins involved in HRR promotion and repression, respectively (135, 141, 142, 185).

ATR recognizes DSBs, but can also be activated by many other DNA damage types. ATR is mainly involved in DNA DSB repair *via* HRR that requires duplicated DNA and occurs in S-phase cells with stalled replication forks or after the G2 phase. ATR recruitment requires single-strand DNA, as observed following DNA DSB end resection by nucleases that are phosphorylated by ATM (*e.g.*, CtIP and exonuclease 1 [EXO1]). Then, there is a progressive switch from the ATM to the ATR signaling cascade. ATR phosphorylates checkpoint kinase 1 (CHK1) that is critical for the intra-S and G2-M checkpoint response and that phosphorylates BRCA1 to trigger HRR (185).

According to this school of thought, DNA DSBs are the central lesions on irradiation and their formation, repair, or nonrepair influences the cellular outcome through complex interplays between cell death and survival signals.

### B. Nuclear-centered view of the cellular response to radiation: conclusion

DDR includes a complex network of proteins activated by DNA lesions, mainly DSBs, produced in irradiated cells. ATM/ATR and MRN play a major role in DDR by recruiting proteins involved in DNA lesion detection and repair, in cell cycle progression, and in cell metabolism (*e.g.*, CHK1, CHK2, p53, BRCA1). However, accumulating evidence indicates that DNA lesions and DDR activation can also be observed in nonirradiated cells close to irradiated cells. This suggests that DDR is transmitted or initiated in these cells (151). We see below (in section IV.A) that the DDR response, specifically ATM, activates the p38 and c-JUN N-terminal kinase (JNK) mitogen-activated protein kinase (MAPK) pathways and also nuclear factor-kappa B (NF-κB) that participates in intercellular signaling.

## IV. Off-Target Effects: An Integrated Cell Response to Radiation

**FIG. 7.**
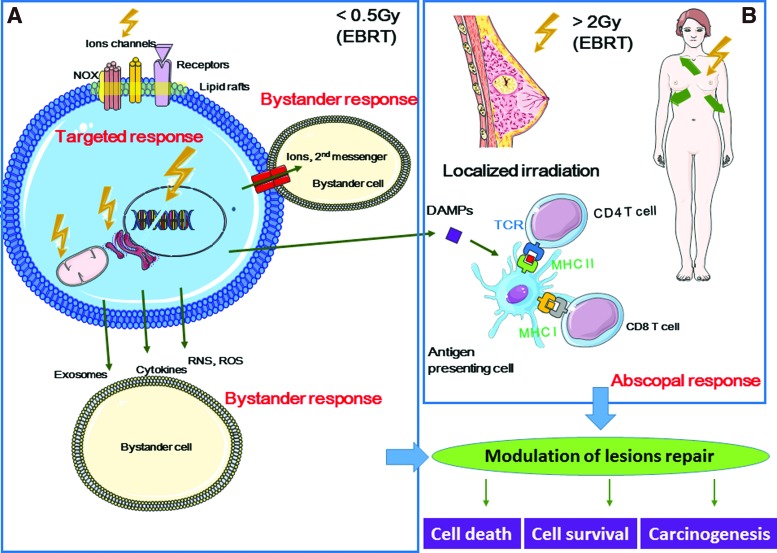
**Schematic representation of the bystander and abscopal responses.** Targeted effects are observed only in irradiated cells, while nontargeted effects are observed in nonirradiated cells. Nontargeted effects include bystander and abscopal responses. **(A)** Radiation-induced targeted effects concern (i) nuclear DNA and extranuclear targets, (ii) mtDNA, Ca^2+^-mediated mitochondrial production of ROS and RNS, (iii) ER, as a Ca^2+^ storage place, and (iv) cell membrane, as the site of ion channels, NADPH oxidase, growth factor and death receptor localization, lipid peroxidation leading to 4-HNE or MDA production, and production of ceramide that acts as a second messenger or is involved in ceramide-enriched large platforms (lipid rafts). Bystander effects are observed in neighboring cells (in contact or not with the irradiated cells) that have not been crossed by ionizing particles. Intercellular cross talk is mediated by gap junctions (GJIC) or through the release of soluble factors, including cytokines, ROS, and RNS. Exosomes containing mRNA, microRNA, and DNA can also be released. **(B)** Abscopal effects are observed at long distance from the irradiation site (*e.g.*, localized breast irradiation). Consequently, the biological effects must be investigated at the whole-body scale. Abscopal effects may involve the immune system through the release of DAMPs that are recognized by antigen-presenting cells (*e.g.*, dendritic cells that will present antigenic peptides to CD4 and CD8 T lymphocytes for immune response activation). The cell response to targeted and nontargeted effects can result in cell death, cell transformation, or cell survival and these possible outcomes have to be taken into account in the field of cancer therapy and radiation protection. 4-HNE, 4-hydroxy-2-nonenal; DAMPs, damage-associated-molecular-patterns; EBRT, external beam radiotherapy; ER, endoplasmic reticulum; GJIC, gap junction intercellular communication; mtDNA, mitochondrial DNA; NAD(P)H, nicotine adenine dinucleotide phosphate; NOX, NAD(P)H oxidase; RNS, reactive nitrogen species. To see this illustration in color, the reader is referred to the web version of this article at www.liebertpub.com/ars

According to the United Nations Scientific Committee on the Effects of Atomic Radiation (UNSCEAR) 2006 report, “bystander effect” is “the ability of irradiated cells to convey manifestations of damage to neighboring cells not directly irradiated,” and “abscopal effect” is “a significant response in a tissue that is physically separate from the region of the body exposed to radiation” (314). Similarly, the International Commission on Radiological Protection describes the bystander effect of radiation as the transmission of signals from irradiated to nonirradiated cells in a cell population, leading to biological changes in the recipient cells (133a, 307) (Fig. 7).

Off-target effects also include induced genomic instability. This is the delayed and stochastic appearance of genomic alterations (chromosomal aberrations, gene mutations, and reproductive cell death) in the offspring of irradiated (and also of bystander) cells, although these daughter cells were not irradiated (198, 199, 200, 201).

During the last 25 years, many studies have investigated the molecular mechanisms underlying off-target effects. They showed that off-target effects are the results of a huge dynamic and integrated process initiated in irradiated cells and transmitted to neighboring cells and to some extent to the whole organism through the activation of the immune system.

### A. Bystander effects

Although off-target effects were first described by Parsons in 1954 (202), Nagasawa and Little performed one of the key early studies that highlighted the need to rethink radiobiology by also considering bystander effects. They used very low mean doses of alpha particles (0.31 mGy) to demonstrate that although <1% of cell nuclei were actually hit by radiation, 30% of cells showed an increased frequency of sister chromatid exchanges (205). Since then, many *in vitro* and *in vivo* studies on bystander effects (usually detrimental for the cells) have been performed using different radiation types (low *vs.* high LET) (7, 131, 272, 278), different cell types (normal and tumor cells) (74, 173, 177) and culture systems (two-dimensional and 3D cell cultures) (238), and different animal models (mammals, fish) (16, 153, 183, 292, 331, 339). Bystander effects were observed for different biological endpoints, such as cell death, apoptosis (166, 179), gene mutations (131, 357), cell differentiation (15), radiation-induced adaptive responses (RIARs) (11, 223), senescence (239), cell cycle distribution, gene expression, chromosomal aberrations, and genomic instability (173, 198–200).

These studies showed that bystander effects depend on the radiation dose, dose rate, and LET (104). They proposed that the contribution of bystander effects to radiation-induced biological effects in conventional external beam radiotherapy (EBRT) and also in therapies using heavy ions, protons, or radionuclides (5, 23, 31, 222, 227, 228, 339) should be taken into account for future applications.

Many cellular components and functions contribute to the extranuclear response to radiation. They include ceramide production and lipid raft formation (111), tyrosine kinases such as epidermal growth factor receptor (EGFR) (270), cytoplasmic Ca^2+^ homeostasis mechanisms (304), protein kinase C (PKC), MAPKs, JNKs (297), phospholipase C (PLC), NF-κB-mediated cyclooxygenase-2 (COX-2) and nitric oxide (NO) synthase (NOS) activation, and cytokines.

Approaches for investigating bystander effects rely on conventional broadbeam and microbeam irradiation to selectively irradiate a single cell or its subcompartments while sparing neighboring cells, or on the transfer of conditioned culture medium from irradiated to nonirradiated cells. The main evidence for extranuclear radiation-induced effects came from cytoplasmic irradiation using microbeams (53, 276, 335). Moreover, Gaillard *et al.* showed that depending on the redox status of the irradiated cells, cytoplasm irradiation by particles could also result in nuclear damage at the origin of bystander signals (90).

ROS and reactive nitrogen species (RNS) initiate events in targeted cells and also participate in their propagation through self-sustained production, leading to similarities between bystander effects and inflammation/immune response. Indeed, the so-called damage-associated-molecular-patterns (DAMPs) released by irradiated tissue can be detected by the innate immune system, as observed for pathogen-associated molecular patterns. Released factors are then recognized by Toll-like receptors (TLRs), C-type lectin receptors, nucleotide binding oligomerization domain-like receptors, and cytosolic retinoic acid-inducible gene-like receptors (151) that are expressed at the surface of immune cells.

#### 1. Intercellular communications between irradiated and nonirradiated cells

Bystander effects involve signaling from irradiated cells to nonirradiated cells. The nature of the communications between irradiated and nonirradiated cells depends on whether cells are in contact or not. This cross talk can be mediated by paracrine secretion in the extracellular space of soluble factors *via* hemichannels formed by the transmembrane protein connexins (Cxs) and pannexins (Panxs) (182, 327). Cxs and Panxs are members of the tetraspan family in which proteins are classified according to their molecular weight (25–62 kDa for Cxs and 1–3 kDa for Panxs). These proteins allow the passage of ions (Ca^2+^, Na^+^) and of low-molecular-weight molecules (nicotinamide adenine dinucleotide, ATP, glutamate, GSH, prostaglandin E2 [PGE2] and inositol trisphosphate [IP_3_]) between the intra- and extracellular environment, thus controlling autocrine and paracrine signaling (68, 327). The role in bystander effects of Cx43, the most abundant Cx in mammals, was identified very early and has been largely investigated (9).

Cxs and Panxs can also form full channels that are called gap junctions and mediate gap junction intercellular communication (GJIC). These intercellular channels show much higher selectivity and allow direct diffusion of factors between the cytoplasm of adjacent cells. The factors involved in bystander effects and transiting through intercellular gap junctions are small molecules (<1.5 kDa) and include ROS (9, 10), RNS (187), ions (Ca^2+^, K^+^, Na^+^), (178, 279), lipid peroxides (83), ATP (221), cyclic adenosine monophosphate (cAMP), glucose, glutamate, and GSH. As hemichannels are less selective than full gap junctions, it has been suggested that in normal conditions only GJIC plays a role. Conversely, in conditions of oxidative stress (*e.g.*, on irradiation), both channel types might be involved (224, 279).

#### 2. Reactive oxygen and nitrogen species initiate and propagate bystander effects

Early experiments highlighted the multiple roles of ROS and RNS in bystander effects. Specifically, they showed that radical scavengers (ascorbic acid, N-acetyl l-cysteine), NOS inhibitors (NG-nitro-l-arginine methyl ester [L-NAME]), NO scavenger (2-4-carboxyphenyl-4,4,5,5-tetramethylimidazoline-1-oxyl-3-oxide [c-PTIO] (281), antioxidant enzymes (superoxide dismutase [SOD], catalase) (10, 303), and DNA-binding antioxidants (methylproamine) (39) inhibit the appearance of bystander effects. For example, genomic instability due to bystander effects can be rescued by restoring mitochondrial function through overexpression of manganese-dependent superoxide dismutase (MnSOD) (103).

**FIG. 8.**
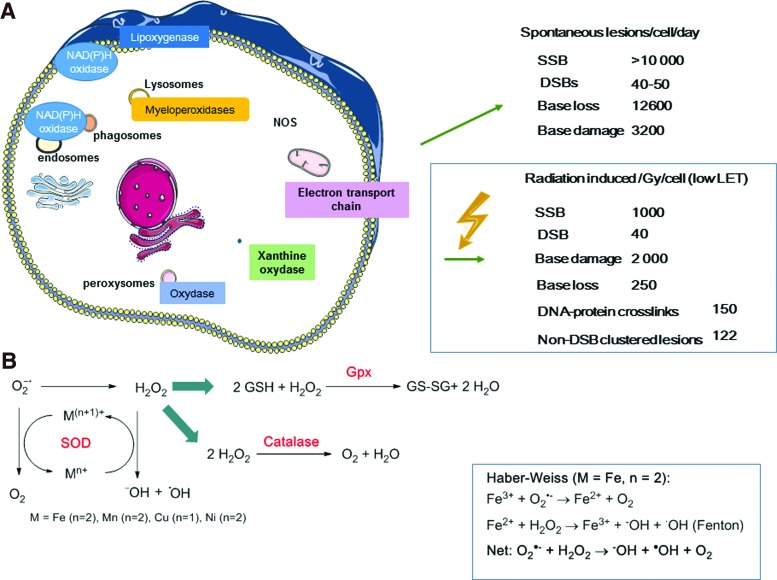
**Endogenous sources of ROS and enzymatic antioxidant defenses**. **(A)** O_2_^−•^, hydroxyl radical (HO^•^), and H_2_O_2_ are produced by endogenous sources that reduce O_2_. The main sources are mitochondria through ATP production and oxidative (oxygenase, dehydrogenase, oxidase) enzymes, such as NAD(P)H, and xanthine oxidase, lipoxygenase, myeloperoxidase. Oxygenase enzymes (*e.g.*, lipoxygenase) oxidize substrates by transferring one electron, while oxidizing a cofactor [*e.g.*, NAD(P)H] in the presence of oxygen. Dehydrogenases use organic substrates as an electron acceptor (*e.g.*, quinones, NAD^+^). Oxidases just use O_2_ as an electron acceptor. The yield of radiation-induced DNA lesions (/Gy/cell) is rather low compared with that produced by endogenous stress [averaged values from Goodhead (102), Pouget *et al.* (241, 245), Sage and Shikazono (262), and Ward (329)]. **(B)** Superoxide can be dismuted into H_2_O_2_ by the action of superoxide dismutase enzymes that possess a metal transition ion (Mn^3+^, Cu^2+^, Fe^3+^, or Ni^3+^) to catalyze the reaction. In the presence of M^(n)+^ metal ions, the resulting H_2_O_2_ can be broken down into HO^•^+OH^−^ and M^(n+1)^, according to the Fenton reaction. The latter reaction can also be mediated by catalase and GPx (22, 76, 80). GPx, glutathione peroxidase; NOS, nitric oxide synthase; SOD, superoxide dismutase. To see this illustration in color, the reader is referred to the web version of this article at www.liebertpub.com/ars

Oxidative processes that involve ROS and RNS continuously occur in cells because at low concentrations (0.01–0.001 n*M* for O_2_^−•^ and 1–100 n*M* for H_2_O_2_), these molecules contribute to essential cellular functions (Fig. 8). At higher concentrations, they could be involved in the defense mechanisms against pathogens and they cause endogenous oxidative damage (22). It is thought that between ten and fifty DNA DSBs are produced per cell each day due to natural ionizing radiation, ROS, DNA replication errors, and unintended cleavage by nuclear enzymes (52, 262). In the cell, the concentrations of free radicals (O_2_^−•^, HO^•^, NO^•^) and nonradical reactive species (H_2_O_2_, ONO_2_) are controlled by the balance between their production and clearance rates that are regulated by antioxidant compounds and enzymes, such as SOD, glutathione peroxidase (GPx), and catalase, as well as nonenzymatic compounds, such as α-tocopherol (vitamin E), β-carotene, ascorbate (vitamin C), and the cellular thiol/disulfide systems that include GSH/GSSH, thioredoxin (TRX1 [-SH2/-SS-]), and cysteine/cystine. Antioxidants can compete for oxidation with oxidizable substrates (at low concentration) and ultimately delay or inhibit the oxidation of these substrates (114). It must be noted that free amino acids, peptides, and proteins, some of which are present at high concentrations in cells (cysteine, tryptophan, histidine, tyrosine), also act as ROS scavengers (80). Reactions between ROS and redox active amino acid residues (*e.g.*, cysteine) can modulate the activity of transcription factors (AP-1, NF-κB, and hypoxia-inducible factor 1 [HIF-1]) and enzymes (*e.g.*, protein tyrosine phosphatases, acid sphingomyelinase [ASMase]) (168).

O_2_^−•^ is one of the main endogenous cellular ROS compounds (Fig. 8). It is produced by reduction of ground-state molecular oxygen (^3^O_2_) by enzymatic (nicotine adenine dinucleotide phosphate [NAD(P)H], xanthine oxidase) or nonenzymatic (semi-ubiquinone Q^−^, a compound of the mitochondrial electron transport chain [ETC]) systems (80).

Peroxisomes also contain enzymes, namely d-amino-acid-oxidase that contains flavin adenine dinucleotide (FAD) as cofactor and urate-oxidase oxidizing substrate by transferring H to O_2_, leading to the formation of H_2_O_2_.

O_2_^−•^concentration is also regulated by three SODs, namely the cytoplasmic Cu–Zn-dependent superoxide dismutase (CuZnSOD or SOD1), the mitochondrial MnSOD (or SOD2), and the extracellular SOD (ECSOD or SOD3), that catalyze O_2_^−•^dismutation into the less reactive H_2_O_2_. This reaction requires reduced transition metals, such as iron or copper ions, and leads to the formation of the precursors of the highly damaging hydroxyl radicals (HO^•^), according to the Fenton reaction (Fig. 8). In cells and in mitochondria, H_2_O_2_ can be decomposed into H_2_O and O_2_ by GPx, catalase, or peroxiredoxins (Fig. 8).

The initial production of ROS by water radiolysis is limited in time, space, and quantity. If one considers that 2000 ionization events are produced per cell per Gy (for low LET radiation), clinical doses of 2 Gy produce much less ROS than the amount generated during standard cell metabolism (329). For instance, a dose of 1 Gy would produce 1 × 10^3^ DNA breaks per cell (about 1000 SSBs and 40 DSBs) (102, 241, 245), while endogenous metabolism would produce more than 10 × 10^3^ DNA breaks per day (262, 318).

Therefore, radiotherapy efficacy in tumor cells can be partly explained by the amplification and prolongation in time (hours and weeks) of the initial radiation-induced ROS production. This would involve ROS- and RNS-mediated activation of signaling pathways and signal transmission to neighboring cells. The main sources of endogenous ROS that participate in this amplification process include mitochondria, NAD(P)H oxidases, and other oxidases, such as xanthine oxidase, lipoxygenases, and peroxisomes (80).

##### a. Mitochondria-dependent ROS production

Human-hamster hybrid AL cells with normal (ρ^+^) or depleted (ρ^0^) mtDNA were used to demonstrate the contribution of mitochondrial functions in the generation of bystander signals from irradiated cells (53, 127, 355).

###### (1) Mitochondria and ROS endogenous production

**FIG. 9.**
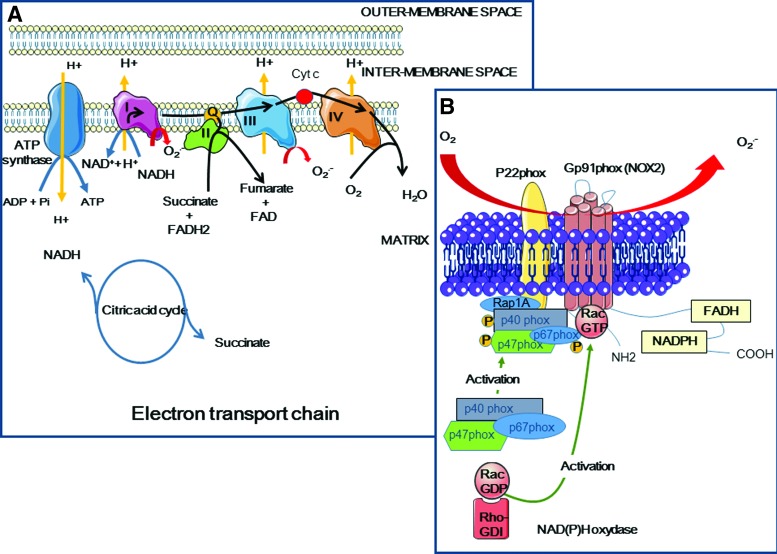
**Mitochondrial electron transport chain and NAD(P)H oxidase as endogenous source of ROS. (A)** NADH and succinate produced during the citric acid cycle are used as electron donors for ATP synthesis by the ETC. ETC consists of complex I (NADH coenzyme Q reductase), complex II (succinate dehydrogenase coenzyme Q), complex III (coenzyme Q cytochrome C reductase), and complex IV (cytochrome C oxidase). The final acceptor molecule O_2_ is reduced to H_2_O. However, a small percentage of electrons can leak at complex I and complex III and can reduce O_2_ into O_2_^−•^. **(B)** The second major source of ROS is NOX. NOX contains membrane proteins (gp91^phox^ or NOX-2 and p22^phox^ that constitute the flavocytochrome b558, and the small G Rap1A protein). During NOX activation (for instance, in neutrophils, or by Ca^2+^ or radiation), cytosolic proteins (p40^phox^, p47^phox^, p67^phox^ and G Rac2) are recruited to the membrane and NADPH binds to NOX and transfer electrons to FAD and further across the membrane to O_2_ (108, 208). ETC, electron transport chain; FAD, flavin adenine dinucleotide. To see this illustration in color, the reader is referred to the web version of this article at www.liebertpub.com/ars

Mitochondria produce energy through the oxidative metabolism of carbohydrates, lipids, and amino acids that generate NADH *via* the ETC. They are also in charge of different metabolic processes, such as the synthesis of heme, nucleotides, lipids, and amino acids. They mediate the intracellular homeostasis of inorganic ions. The ETC is composed of four multisubunit enzyme complexes (Fig. 9): complex I with NADH coenzyme Q reductase activity, complex II with succinate dehydrogenase coenzyme Q activity, complex III with coenzyme Q cytochrome C reductase activity, and complex IV with cytochrome C oxidase activity. Coenzyme Q (or ubiquinone) allows the electron transfer from complex I to III, while cytochrome C is involved in the transfer between complex III and IV. Electrons are finally transferred to O_2_, leading to water formation. Intermediate products, such as O_2_^−•^ and H_2_O_2_, may also be liberated, particularly at complex I (NADH dehydrogenase) (107) and III (310), during or after premature leakage of electrons between ubiquinone and cytochrome C, and at complex II, to a lower extent (151, 348).

###### (2) Mitochondria and irradiation

Mitochondria are essential in the intrinsic apoptosis pathway (but not in the death receptor-mediated extrinsic pathway). Specifically, mitochondrial membrane permeabilization and cytochrome C release are followed by caspase activation. The intrinsic apoptosis pathway involves PUMA, a proapoptotic protein that allows BAX and/or BAK translocation to mitochondria to signal apoptosis.

The total cellular mitochondrial volume is high (4–25% of the cell volume, depending on the cell type and state), and therefore, mitochondria represent a fairly substantial target for cytoplasmic irradiation. Radiation produces many different types of mitochondrial damage, including mtDNA damage. mtDNA is a circular double-stranded genome that encodes proteins, transfer RNA, and ribosomal RNA. It lacks protective histones and shows limited repair capability. Therefore, on radiation exposure, mtDNA could be preferentially damaged or lost (217), leading to loss of mitochondrial function (149, 316). Moreover, it has been shown that microbeam irradiation with carbon ions leads to depolarization of mitochondria (323). Exposure to direct gamma radiation causes mitochondrial mass increases (218), and alpha-particle microbeam irradiation leads to mitochondrial fragmentation, involving dynamin-related protein 1 (DRP1), a member of the dynamin family involved in mitochondrial fusion and fission (348).

Cytoplasmic irradiation also has been associated with altered protein synthesis and irregular mitochondrial oxidative phosphorylation (66, 217, 348), leading to persistent oxidative stress (162, 345). Yoshida *et al.* detected dysfunction of complex I at 12 h postgamma irradiation, after early and transient production of intracellular ROS (in the first minutes). This was followed by increased mitochondrial ROS levels and mtDNA damage at 24 h (345). These observations suggest that mitochondria participate in the amplification of the initial oxidative stress through transmission of oxidative events from one mitochondrion to the entire mitochondrial population of a given cell. This process might involve transient Ca^2+^-dependent mitochondrial permeability to adjacent mitochondria, resulting in enhanced ROS/RNS generation (53, 162).

Chen *et al.* showed that in mtDNA-depleted and wild-type AL cells treated with mitochondrial respiratory chain function inhibitors, γH2AX induction is attenuated in bystander cells (53). Incubation of HepG2 (liver cancer) cells with cyclosporin A, an inhibitor of cytochrome C release, before irradiation demonstrated that cytochrome C release regulates bystander effect-induced formation of micronuclei (MN) and production of NO, but not of ROS (117). This indicates that cytochrome C has an effect on inducible NOS (iNOS) that catalyzes NO production.

Zhou *et al.* showed that on alpha-particle microbeam irradiation, the frequency of DNA mutations is decreased in bystander cells in cultures of skin fibroblasts with reduced (ρ^0^) mitochondrial functions compared with wild-type (ρ^+^) cells. Moreover, incubation with a pharmacologic inhibitor of NF-κB activation (Bay 11-7082) or with the NO scavenger c-PTIO reduced the DNA mutation frequency in both mutant (ρ^0^) and wild-type (ρ^+^) cells, highlighting the role of NF-κB and of activation of its downstream NF-κB/iNOS and NF-κB/COX-2 signaling pathways (355).

Finally, mitochondria are affected also in nonirradiated cells grown in culture medium from irradiated cells. Specifically, changes of mitochondrial distribution, loss of mitochondrial membrane potential, and mitochondrial mass (218), increased ROS and RNS production, and increased apoptosis rates have been observed in such cells. These effects can be blocked by antioxidant treatments (180).

##### b. NAD(P)H oxidase-dependent ROS production

The plasma membrane-bound NAD(P)H oxidase (NOX) is another endogenous source of ROS. It is found in phagocytic cells, such as macrophages and neutrophils (innate immunity), where it was first identified (14, 259). NOX-1 participates in pathogen killing *via* the formation of O_2_^−•^ by transferring one electron from NADH or NADPH (Fig. 9) (80), which are located on the cytoplasmic face of the plasma, endosomal, or phagosomal membranes, to O_2_, which is found in the extracellular space, or in the lumen of endosomes and phagosomes. O_2_^−•^ can next dismute spontaneously or *via* peroxidase into H_2_O_2_ that can be transformed into hypochlorous acid (HOCl) and contribute to the antimicrobial activity of neutrophils (208).

NOX includes several subunits, such as membrane proteins (gp91^phox^ or NOX-2 and p22^phox^ that constitute flavocytochrome b558, and the small G Rap1A protein) and cytosolic proteins (p40^phox^, p47^phox^, p67^phox^, and the G Rac2 protein). Flavocytochrome b558 contains heme subunits with low oxydo-reduction potential (−225 and −265 mV at pH 7.0) and is mainly involved, *via* its p67^phox^-mediated activation, in O_2_ reduction by binding to NADPH and FAD (14). An electron chain transport then occurs from NADPH to O_2_ *via* FAD and heme (108). Six human NOX isoforms have been identified in nonphagocytic cells. NOX-1, NOX-3, and NOX-4 show similarities with NOX-2, while the DUOX (*dual oxidase*) group includes DUOX-1, DUOX-2, and NOX-5. DUOX-2 peroxidase activity leads to H_2_O_2_ formation and is involved in the biosynthesis of thyroid hormones. NOX proteins play different roles according to the cell types where they are expressed and to the levels of ROS production (14).

In irradiated fibroblasts, NOX activation does not require direct nuclear or cellular “hits” by alpha particles and leads to O_2_^−•^ and H_2_O_2_ generation, and also to persistent ROS production in bystander cells (207). NOX is located in ceramide-enriched lipid raft domains, the disruption of which leads to NOX activity inhibition (347). For instance, transforming growth factor (TGF) β1 secreted by irradiated cells activates NOX-1 and the subsequent O_2_^−•^ production in bystander cells (277). Moreover, TGFβ1-mediated activation of DUOX proteins and the release of the peroxidase domain by metalloproteases (1) are required for the HOCl signaling pathways. O_2_^−•^ production by NOX might participate in the activation of ASMase, leading to NOX activation in ceramide-enriched domains (337). It was shown that irradiation induces NOX/DUOX-1-dependent H_2_O_2_ production for several days (6). This process involves p38 MAPK-mediated activation of NOX *via* IL-13 expression. NOX can then promote the amplification and long-term persistence of oxidative stress signals (6).

##### c. ROS and RNS as second messengers

Most ROS, such as HO^•^, are very reactive and have a very short life, and thus react within a few nm range from their site of production. They cannot be transmitted to neighboring cells. However, H_2_O_2_ and NO can diffuse through the cell membrane. H_2_O_2_ can cross membranes through aquaporin channels (21) and can diffuse through the cytoplasm and plasma membranes of neighboring cells along several cell diameters (13, 340). NO, one of the main RNS, can damage DNA (211), is mutagenic, and is involved in proapoptotic signal transduction (211). NO also plays a role in blood compartment functions, including smooth muscle tone and blood pressure regulation, platelet activation, and vascular cell signaling. Thanks to its lipophilic properties and relative stability, NO can activate signaling processes in adjacent cells (161).

NO is upregulated on oxidative stress and therefore can compete for substrates with SOD by reacting with O_2_^−•^ to form the diffusible peroxynitrite (ONOO^−^) (13, 340). In agreement, incubation with L-NAME (an NOS inhibitor), but not with rotenone (an inhibitor of electron entry into complex I of the mitochondrial ETC), leads to an increase of oxidative stress. This suggests that constitutive levels of NO production contribute to the regulation of mitochondrion-derived intracellular oxidant generation (99). When NO concentration increases to the level of SOD, ONOO^−^ can rearrange into biologically inert nitrite (NO_2_^−^) or react with GSH to form the NO donor GSNO (161). However, it can also spontaneously and rapidly decompose to nitrogen dioxide (NO_2_) and HO^•^, thereby oxidizing lipids, thiols, amino acid residues, DNA bases, and low-molecular-weight antioxidants, as done by ROS cellular constituents (13). NO can also modify proteins, leading to S-nitrosylation (80, 164) or nitration, mainly tyrosine nitration, a marker of tissue inflammation. This posttranslational modification participates in the regulation of cellular functions. For example, NO affects the DNA repair mechanisms by downregulating the expression of BRCA1, involved in HRR and cell cycle checkpoint control, while promoting the error-prone NHEJ mechanisms. This regulation seems to be mediated *via* tyrosine nitration of protein phosphatase 2A (PP2A). PP2A then controls the formation of the retinoblastoma-like protein 2 (RBL2)/E2F4 inhibitory complex that recognizes and binds to the proximal BRCA1 promoter (341).

NO production from l-arginine is catalyzed by one of the NOS isoforms, such as constitutive NOS (cNOS), including neuronal NOS (nNOS or NOS1) and endothelial NOS (eNOS or NOS3) and iNOS (known as NOS2) that are present in the cytoplasm and subcellular organelles. Different from NOS and eNOS, iNOS activation is independent of Ca^2+^. Irradiation stimulates cNOS transient activation with a maximal activity 5 min after exposure to clinical doses of 2 Gy (161).

Pretreatment with the NO scavenger c-PTIO of cells exposed to microbeam with alpha particles abolishes MN formation in bystander cells (280). Moreover, incubation of a mouse leukemic monocyte macrophage cell line (RAW 264.7) with lipopolysaccharide (LPS) induces iNOS activity and NO generation, thereby increasing DNA damage in bystander EL-4 lymphoma cells (97).

#### 3. Cell membrane response to radiation

Compared with DNA-centered approaches, relatively few radiobiological studies have investigated the role of radiation targeted at the cell membrane that, for long time, was considered to be just an inactive phospholipid bilayer. However, in the 1990s, several studies showed the production of ceramide, a cellular second messenger of apoptosis involved in the sphingomyelin signaling pathway, during membrane irradiation (111). Using lymphoblasts from patients with Niemann–Pick disease (deficiency in ASMase activity), it was demonstrated that this enzyme is required for radiation-induced production of ceramide and apoptosis (263). Many studies now strongly support the ceramide-mediated cell membrane role in the biological effects of radiation, including cell death. Moreover, radiation resistance in Burkitt's lymphoma cells has been associated with defective ceramide signaling (189). Finally, structure-modulating agents (*e.g.*, cholesterol) and antioxidants (*e.g.*, tocopherol, eugenol) can modify the membrane response to stress (194, 230).

##### a. Cell membrane and lipid rafts

Cell membrane contains lipids (sphingolipids and glycerophospholipids), proteins, and sterols (Fig. 5B).

Lipids, such as PUFA, are susceptible to free radical-initiated oxidation (49), leading to lipid hydroperoxide formation that can then be reduced by peroxidases. If not efficiently reduced, lipid hydroperoxides can be degraded into hydroxyalkenals (such as 4-HNE) that show great reactivity toward DNA, proteins, and lipids (49).

Cholesterol and sphingolipids, among which sphingomyelin is the prevalent, mainly localize in the outer leaflet of the cell membrane. They play a crucial role in signal transduction and participate in cell growth, senescence, differentiation, and apoptosis (122). Sphingolipids contain a long-chain sphingoid base (such as sphingosine) linked *via* an amide to long-chain fatty acids and to one polar head group, making them amphipathic molecules (Fig. 5B). Head groups are different in sphingolipids (phosphorylcholine), ceramides (hydroxyl group), and glycerophospholipids (carbohydrates) (256). Ceramide is composed of D-erythro-sphingosine and of a fatty acid that contains 2–28 carbon atoms in the acyl chain. Similarly, a hydrophobic moiety forms the backbone of sphingomyelin and of other complex sphingolipids, such as cerebrosides and gangliosides.

The concept of the cell membrane as a fluid mosaic (289) is based on the finding that most physiological phospholipids exhibit low melting temperatures and, therefore, most likely exist in a liquid disordered phase. For instance, in *Escherichia coli*, the fatty acid composition of phospholipids depends on the temperature. Specifically, the proportion of unsaturated acids increases as the temperature decreases (67). At physiological temperature (37°C), 22% of the phospholipid molecules have two unsaturated acyl chain molecules, whereas this proportion is two times higher at 17°C (67).

However, the notion of fluid mosaic has been reconsidered because mammalian membranes contain very small domains that are in a liquid ordered phase (35, 287). Indeed, sphingolipids, which have a much higher melting temperature than other phospholipids in the cell membrane, interact with each other *via* hydrophilic interactions between the sphingolipid head groups. These complexes are stabilized by cholesterol that fills the gaps between the large sphingolipid molecules. The resulting domains are resistant to cold detergent extraction (2) or mechanical disruption and are called lipid rafts because they seem to float in the membrane. With a size of about 50 nm, they correspond to lateral subcompartments in the cell membrane and allow the cell membrane to exert its cellular functions by segregation of molecules, reorganization of receptor molecules and of membrane signaling and trafficking.

**FIG. 10.**
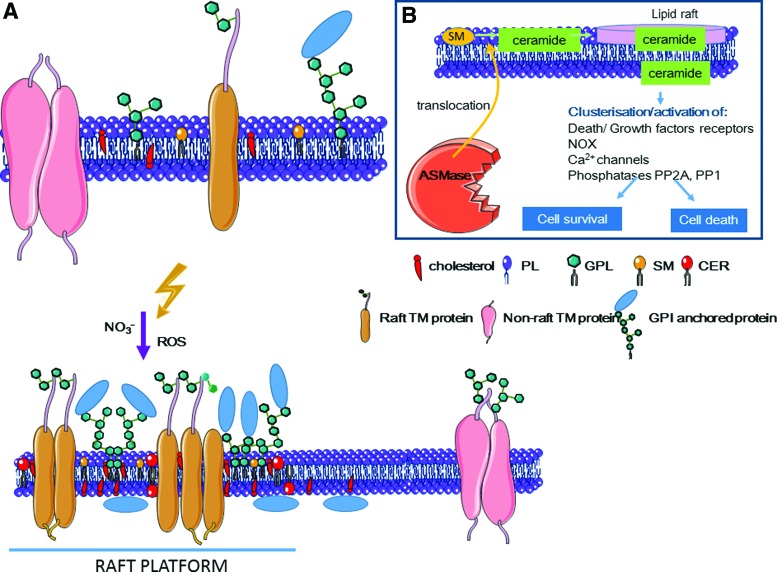
**Lipid rafts: ceramide-enriched large platforms**. **(A)** The cell membrane consists of a lipid bilayer that includes proteins and cholesterol. According to the fluid-mosaic model, lipids can rotate laterally and between bilayers. The lipid distribution in the cell membrane is associated with specific cellular functions. Specifically, the cell membrane can include glycolipids, phospholipids (*e.g.*, phosphatidylcholine, phosphatidylserine, phosphatidylethanolamine), and sphingolipids, among which SM is predominant. Their resistance to disruption allowed the identification of membrane domains enriched in ceramide. Ceramide is produced *via* ASMase-mediated hydrolysis of SM following RNS and ROS activation and ASMase translocation to the outer layer of the cell membrane. Interaction between the resulting lipids and proteins leads to the coalescence of microdomains (lipid rafts) into ceramide-enriched large platforms. **(B)** These platforms can promote clustering of receptors and activation of signaling pathways. Besides its role in lipid rafts, ceramide is also a second messenger (58, 167, 346). CER, ceramide; GPL, glycophospholipids; TM, transmembrane. To see this illustration in color, the reader is referred to the web version of this article at www.liebertpub.com/ars

Lipid rafts can be converted into larger membrane platforms by ASMase activity that hydrolyzes sphingomyelin to ceramide in rafts (Fig. 5B). Ceramide molecules then spontaneously associate to form ceramide-enriched microdomains that fuse into large ceramide-enriched membrane platforms, thus altering the biophysical properties of these membrane domains (167) (Fig. 10). ASMase is activated by multiple stimuli, including CD95, CD40, DR5/TNF-related apoptosis-inducing ligand (TRAIL), CD20, FcgRII, CD5, LFA-1, CD28, cytokines, and chemotherapeutic drugs (doxorubicin, cisplatin), and also by ionizing radiation. Ceramide is considered the second messenger of many factors. For instance, ceramide acts as a messenger of inflammatory cytokines by binding and exerting a dual effect on the cytosolic phospholipase A2 (cPLA2) involved in eicosanoid formation (132). Ceramide is also involved in apoptosis. Its binding to the endosomal acidic aspartate protease cathepsin D results in the autocatalysis of the 52 kDa prepro-cathepsin D into the enzymatically active 48/32 kDa cathepsin D isoforms that mediate oxidative stress-induced apoptosis (120).

It has been proposed that oxidation of cysteine 629 in ASMase C-terminus by HO^•^ could be responsible for a possible mechanism leading to increased ASMase enzymatic activity (251). Moreover, the RNS ONOO^−^ specifically activates ASMase (48).

Ceramide is generated by ASMase in the outer leaflet of the cell membrane or within intracellular vesicles. It might also flip to the cytoplasmic leaflet and then interact with intracellular molecules. Although the mechanisms of ceramide translocation from one membrane leaflet to another are unknown, ceramide can interact and activate serine/threonine phosphatases, namely PP2A and PP1 (50, 58) (Fig. 10). Once activated, these phosphatases act on different signaling proteins, including MAPKs (AKT, c-JUN), PKC isoforms (PKCα and ζ), kinase suppressor of Ras (KSR), pRB, and BCL-2 (28, 349).

##### b. Lipid rafts in mitochondria-ER-associated domains

Several recent studies indicate that ceramide is also present in mitochondria (122). Mitochondrial ceramide could be generated *via* the *de novo* synthesis pathway through reverse activity of ceramidase and activity of ASMase, residing in the space between the inner and outer mitochondrial membrane. Lipid raft domains might be involved in contact regions between ER and mitochondria, leading to the direct transfer of Ca^2+^ released from ER to mitochondria (98). It has been reported that mitochondria-ER-associated domains contain ROS generating proteins and IP_3_ receptors (98). These receptors are upregulated by irradiation and are involved in ATP-mediated Ca^2+^ release from ER (343). Ca^2+^, NO, ROS, and molecules modulated by irradiation, such as AKT, promote IP_3_ receptor expression. For instance, it was shown that inhibitors of PI3K, an upstream AKT activator, can block Ca^2+^ release from ER (342).

##### c. Caveolae, a subgroup of membrane lipid rafts

Caveolae are a subset of membrane lipid rafts that make small invaginations in the plasma membrane, ER, and Golgi apparatus. They contain caveolins that act as organizing centers for cellular signal transduction (233). Caveolin-1 contains a tyrosine 14 phosphorylation site and its phosphorylation leads to its accumulation at focal adhesion sites (*i.e.*, mechanical and biochemical hubs between cells and extracellular matrix) and to the subsequent transmission of extracellular signals *via* intracellular pathways (118). Caveolin-1 phosphorylation at tyrosine 14 by the proto-oncogene tyrosine protein kinase Src is involved in EGFR and AKT activation. Caveolins serve as scaffolding proteins and can dock signaling molecules, such as the Src kinase, PI3K, eNOS, protein kinase A, PKC, and extracellular signal-related kinase (ERK). The interaction between caveolin-1, beta1 integrin, and focal adhesion kinase (FAK) enhances cell adhesion and radioresistance by promoting phosphorylation of AKT and GSK-3beta, two factors involved in prosurvival pathways (118).

##### d. Ion channels and lipid rafts

Ceramide-enriched membrane platforms are involved in the regulation of potassium (27) and calcium (56) channels. Calcium ion cytoplasmic level is critical for many cellular functions *via* interaction with various signaling cascades, such as those that involve the activation of the calcium-dependent PKC (112) or Ca^2+^/calmodulin kinase (81).

Intracellular Ca^2+^ can activate NOX and iNOS, thereby promoting ROS and RNS formation, respectively. It can also modulate the activity of the transcription factors NF-κB and AP1 that are involved in COX-2 and iNOS transactivation and the subsequent release of ROS, RNS, and cytokines. Moreover, it activates kinases, including PI3K/AKT and MAPK.

**FIG. 11.**
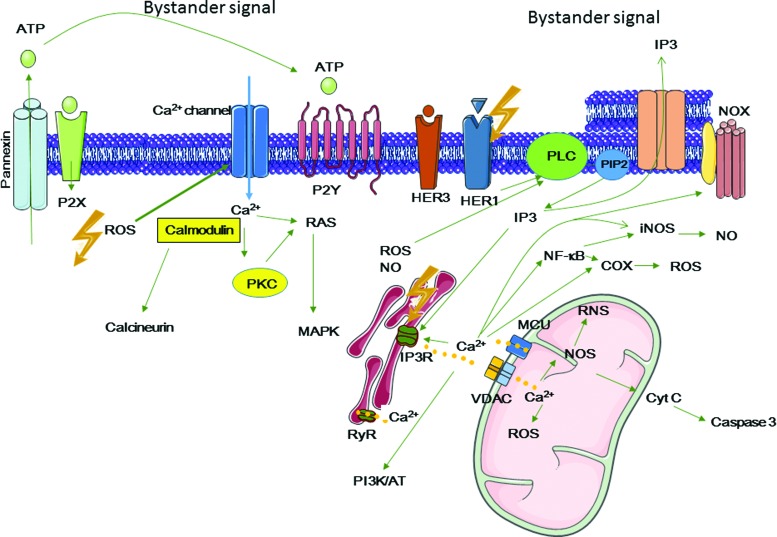
**Interplay between Ca^2+^ and radiation-induced oxidative stress [extensively reviewed in Decrock** ***et al.*** **(68)].** Irradiation increases the intracellular Ca^2+^ level (oscillations or single transient changes occurring within minutes to days after irradiation). Radiation-induced ATP release by irradiated cells can activate ATP-gated P2X receptor cation channels (P2X receptors) present on the cell membrane, thus allowing Ca^2+^ entry into the cell. It can also activate P2Y receptors that have been identified as phospholipase C activators. Ca^2+^ can also be released from the ER through calcium-induced calcium release mechanisms that involve IP_3_Rs or RyRs. IP_3_ is produced (with diacylglycerol) during hydrolysis of phosphatidylinositol 4,5-bisphosphate (PIP2) by phospholipase C. Phospholipase C is activated by Ca^2+^, GPCRs, ROS, RNS, receptor and nonreceptor tyrosine kinase (*e.g.*, HER1 and 3). Ca^2+^ can activate ion channels and binds to calmodulin before activation of the serine/threonine protein phosphatase calcineurin. Ca^2+^ can also activate protein kinase C that in turn activates, by phosphorylation, the MAPK pathway and phospholipase A2, at the origin of COX-2 activity modulation. It can activate transcription factors (NF-κB, AP1) that promote various downstream pathways (iNOS, COX-2). Released Ca^2+^ can also be taken up by mitochondria *via* VDAC and MCU and modulation by the ER-mitochondria-tethering proteins GRP75 (mitochondrial heat shot protein HSP70) and MFN 1 and 2 (involved in mitochondrial fusion). The increase in mitochondrial Ca^2+^ level is accompanied by ROS, an RNS increase, mtDNA damage, altered ATP synthesis, mitochondrial depolarization, and release of cytochrome C and caspase 3 that will amplify IP_3_R activity. COX-2, cyclooxygenase-2; GPCRs, G-protein-coupled receptors; iNOS, inducible nitric oxide synthase; IP_3_, inositol trisphosphate; IP_3_Rs, IP_3_ receptors; MAPK, mitogen-activated protein kinase; MCU, mitochondrial Ca^2+^ uniporter; MFN, mitofusin; NF-κB, nuclear factor kappa B; RyRs, ryanodine receptors; VDAC, voltage-dependent anion channel. To see this illustration in color, the reader is referred to the web version of this article at www.liebertpub.com/ars

In response to irradiation, Ca^2+^ levels increase (112, 319, 352). These changes can be described as oscillations or single transient changes within minutes to days after irradiation. The concentration of free Ca^2+^ in the cytoplasm is low (∼100 n*M*) in normal physiological conditions. It is controlled by modulating its cellular entry from the extracellular environment through, for example, ROS-mediated activation of plasma membrane Ca^2+^ channels, or by release from internal storages where it is present at higher concentrations (up to m*M*). ER is the main Ca^2+^ store (Fig. 11). As shown by experiments using the PLC gamma inhibitor U73122, Ca^2+^ release is regulated by PLC-mediated generation of the second messenger IP_3_ that binds to the IP_3_ receptors located at the ER membrane (20, 305) (Fig. 11). This process is mediated by activation of plasma membrane-associated receptor tyrosine kinases, such as ERBB3 and HER1. HO^•^ and exogenous NO induce the release of calcium from intracellular IP_3_ receptor-sensitive stores and they modulate the open/closed status of Cx channels. Moreover, ROS can activate PLC (40, 229). An increase in IP_3_ receptor level was observed after a dose of 3 Gy in lymphoblastoid cells (343). Moreover, IP_3_ receptor phosphorylation by AKT is associated with reduced Ca^2+^ flux from the ER to mitochondria and subsequently with reduced apoptosis (299).

##### e. The role of Ca^2+^ ions in bystander effects

Ca^2+^ ion role as second messenger has been highlighted by several studies using calcium chelators or blockage of voltage-gated *L*-type Ca^2+^ channels. A rapid intracellular calcium increase was observed in bystander keratinocytes on addition of medium from X-ray irradiated keratinocytes. This phenomenon was associated with increased ROS production, decreased mitochondrial membrane potential, and apoptosis (178, 279). Moreover, membrane signaling and Ca^2+^ influx induced in bystander cells by ROS-activated signaling factors released from irradiated cells lead to ROS production in bystander cells (176). Several factors could mediate the propagation of the increased intracellular Ca^2+^ levels. Specifically, it was shown that ATP released by irradiated astrocytes is the determinant of calcium wave propagation over large distances (100–250 μm) (30). ATP may act as an extracellular signaling molecule by diffusing *via* channels that involve Cxs (61), Panxs (298), or purinergic P2XR7 receptor channels (221, 306, 311, 313). Once in the extracellular space, ATP can activate Ca^2+^-permeable channels or G-protein-coupled receptors (GPCRs) on neighboring cells, thus inducing PLC-mediated IP_3_ synthesis (68). IP_3_ can also diffuse *via* membrane gap junctions (266) (Fig. 11) and activate PLC in neighboring cells.

Ca^2+^ released from ER can be transferred to mitochondria through voltage-dependent anion channels (VDACs) and mitochondrial Ca^2+^ uniporter (79, 257). Accumulated Ca^2+^ can then activate mitochondrial metabolic functions and stimulate energy production *via* ATP synthase (252), and subsequently ROS and RNS production and release of the proapoptotic factors cytochrome C and caspase 3. Moreover, irradiation can modulate the expression of GRP75 and mitofusin 1 and 2 (MFN 1/2), involved in ER-mitochondrial cross talk. It can also regulate Cx43 and Cx30 translocation to the inner membrane of mitochondria where they participate, as hemichannels, in Ca^2+^ homeostasis (68).

Studies in which human keratinocytes were incubated with conditioned medium from gamma particle-irradiated cells in the presence of EGTA, verapamil, nifedipine, or thapsigargin (known to act on calcium homeostasis) showed the involvement of calcium and the activation of multiple MAPK pathways, such as the ERK, JNK, and p38, in the production of radiation-induced bystander effects (178). Similar results were obtained during *in vitro* radionuclide therapy using Auger electron emitters (228). Other studies reported calcium ion role in NO-mediated bystander effects through calcium-dependent NO generation *via* cNOS (NOS1) or eNOS (NOS3) (115, 279). Inhibition of cNOS leads to a decrease in radiation-induced ERK1/2 kinase activity (161).

##### f. ROS/RNS and growth factor receptor activation

**FIG. 12.**
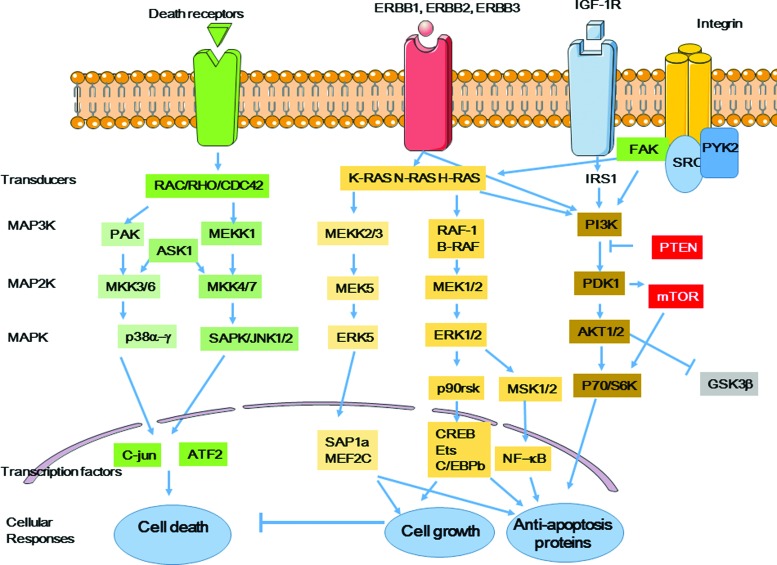
**The MAPK and PI3K signaling pathways**. The four major MAPK signaling pathways (ERK1/2, ERK5, JNK1/2, and p38) regulate cell survival (ERK1/2 and ERK5) and apoptosis (JNK1/2 and p38) *via* the expression of transcription factors. The PI3K signaling pathway is involved in cell growth *via* AKT1/2 proteins. Death (Fas/CD95, TNF-R, DR3-5) and growth (HER family and IGF-1 receptors) receptors are located in lipid rafts and can be activated during coalescence of ceramide-enriched raft platform. They facilitate the cross talk between death or growth signaling from the membrane environment and intracellular signaling cascades. FAK and PYK-2 serve as scaffold proteins to facilitate the functional integration of focal adhesion proteins, such as paxillin, involved in Ca^2+^ homeostasis and can phosphorylate PI3K. ERK, extracellular signal-related kinase; FAK, focal adhesion kinase; IGF-1, insulin-like growth factor 1; JNK, c-JUN N-terminal kinase; PTEN, prime time entertainment network. To see this illustration in color, the reader is referred to the web version of this article at www.liebertpub.com/ars

Lipid raft-mediated membrane reorganization can lead to activation of receptor and nonreceptor tyrosine kinases, such as ERBB1, that can homodimerize or heterodimerize with other members of the ERBB receptor family (ERBB2, ERBB3, and ERBB4) (91), insulin-like growth factor-1 (IGF-1) receptors, and death receptors (Fig. 12). Binding of growth factors to their tyrosine kinase receptors leads to activation of MAPK and PI3K/AKT signaling pathways. These processes are mediated by similar signaling pathways. Indeed, activation of ERBB1, 2, and 3 leads to activation of RAS family members (K-RAS, N-RAs, H-RAS) and further downstream signaling pathways that involve MAP3K (MEKK2/3, RAF-1/B-RAF), MAP2K (MEK1/2; MEK5), and MAPK (ERK1/2) and in turn activate transcription factors, such as AP1, NF-κB, and CREB (315). Similarly, death receptors activate two other MAP3K/MAP2K/MAPK pathways resulting in the activation of p38 (α−γ) kinases and JNKs (1/2). Finally, IGF-1 receptor and its downstream kinases PI3K, PDK1, and AKT participate in mTOR activation and GSK-3 regulation. Ultimately, all these different pathways result in the activation of transcription factors involved in cell death or survival.

Radiation can modulate the production and expression of cytokine and growth factor receptors (54, 270, 305). For example, a 2 Gy dose can modulate the expression of cell surface receptors, such as ERBB1 (HER1) (305). As described above, NO and ROS produced by radiation can lead to defective mitochondrial function due to leakage of mitochondrial membranes, allowing the amplification of the initial signal through massive release into the cytosol, *via* Ca^2+^-dependent mechanisms, of O_2_^−•^ anions and RNS. These, in turn, may inhibit the activity of protein tyrosine phosphatase by oxidation and/or nitrosylation of a key cysteine residue in the active site, leading to increased phosphorylation of many proteins, such as ERBB1 (308). Cells lacking functional mitochondria (ρ^0^ cells) cannot inhibit such phosphatase activities (162). Similar results were reported on ROS scavenging by *N*-acetyl cysteine.

The PI3K pathway, which plays a role in the long-term effects of cell survival, can also be activated on irradiation (315).

It was shown that activation of growth receptors is modulated by radiation according to waves. For example, Dent *et al.* found that EGFR is first activated early and for few minutes (0–5 min) after irradiation. This is followed by a second prolonged activation at 90–240 min postirradiation (73). The MAPK pathways activate membrane-bound matrix metalloproteinase (MMP) activities that promote cleavage of proforms/zymogens of multiple growth factors into their functionally activated forms that can in turn activate, later after irradiation, cell surface receptors. For example, EGFR activation could in turn promote cleavage and release of presynthesized paracrine ligands, such as pro-TGFα (109, 315) that could contribute to sustained MAPK signaling *via* an autocrine positive feedback loop involving EGFR, the Ras-MAPK signaling pathway, and a ligand-releasing protease (284). Released factors could then bind to cells that express EGFR or be transmitted in a paracrine way for long-distance effects (abscopal effects). The observation that a broad-spectrum MMP inhibitor (marimastat, BB2516), known to affect tumor invasion, inhibits also growth of head and neck squamous cell carcinoma (HNSCC) cells that overexpress EGFR, highlighted the MMP role (*e.g.*, MMP9) in cleaving HER ligands from membrane-anchored precursors into their functionally activated states (218a). Inhibition of ERK/MAPK (by PD98059 or U0126) and PI3K (by LY294002 or wortmannin) leads to a marked reduction of both basal and induced MMP9 activity, indicating that the MAPK signaling pathways are also required in an autocrine loop for HER1 activation (218a).

##### g. MAPKs and the bystander response

The four major MAPK signaling pathways in mammalian cells (ERK1/2, ERK5, JNK1/2, and p38) can regulate either survival (ERK1/2 and ERK5) or apoptosis (JNK1/2 and p38). Inhibitors of the ERK pathway (PD98059 and U0126), JNK pathway (SP600125), and p38 pathway (SB203580, SB202190) helped demonstrating the role of these signaling pathways in bystander cell death induced by gamma/X-ray (55, 178), alpha particle (89), or carbon ion (77) exposure. Similar results were obtained after radionuclide therapy with Auger electron (228) or alpha particle-emitters (unpublished data). ROS and RNS have a role in MAPK direct and indirect activation. Incubation of confluent cultures of human diploid fibroblasts with CuZnSOD or catalase after irradiation with 0.003–0.03 Gy of alpha particles inhibits p21 (WAF1) upregulation by MAPK and the induction of MN formation in bystander cells (10). Moreover, p21 knockout results in inactivation of MAPK signal pathway kinases (55), and cNOS inhibition leads to reduction of ERK1/2 kinase activity (cytoprotective effect) (161), suggesting that diffusible NO could mediate MAPK activation in bystander cells.

#### 4. Central role of NF-κB in the nuclear and extranuclear responses to radiation

Experiments showing that ATM activates NF-κB, which is involved in iNOS and COX activity, demonstrated the link between inflammation and DDR (336). Therefore, as the bystander and inflammatory responses share homologies, studying the role of NF-κB in bystander effects can be of major relevance.

##### a. Nuclear factor kappa B

**FIG. 13.**
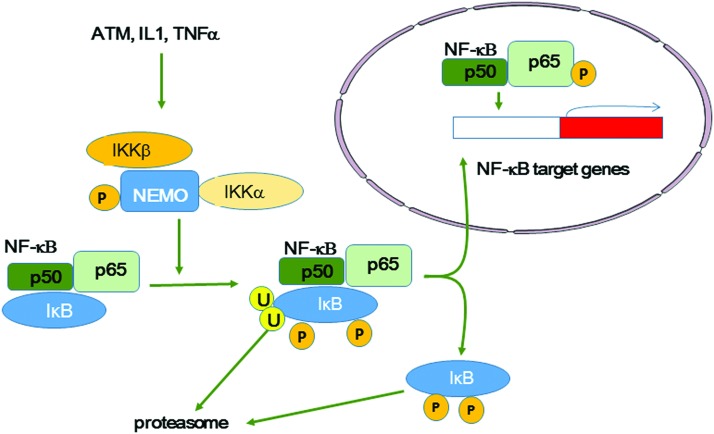
**NF-κB activation mechanisms.** The antiapoptotic NF-κB consists of five heterodimers, among which the most common form involves p65 and p50. It is kept in the cytoplasm by its interaction with IκB. On activation (*e.g.*, by ATM, TNFα, IL1), the IKK complex, which is made of IKK-α (IKK1), IKK-β (IKK2), and the regulatory subunit IKK-γ/NEMO, phosphorylates and targets IκB for ubiquitination and degradation by the proteasome, while NF-κB can enter the nucleus and activate its target genes. IKK, IκB kinase; NEMO, NF-κB essential modulator. To see this illustration in color, the reader is referred to the web version of this article at www.liebertpub.com/ars

NF-κB is a redox-sensitive transcription factor made of five different subunits that are organized in homo- or heterodimers: p50 (*e.g.*, p50/p105 constitutes NF-κB1), p52 (*e.g.*, p52/p100 constitutes NF-κB2), p65^RelA^, RelB, and c-Rel (Fig. 13). All subunits have a 300-amino acid N terminal sequence (Rel Homology Domain) that mediates dimerization, nuclear translocation, DNA binding, and I-κB binding. NF-κB is involved in innate and adaptive immunity and chronic inflammation, thereby linking the immune response to radiation response (121). Moreover, it is a transactivator of genes involved in cell proliferation and suppression of apoptosis induced by tumor necrosis factor (TNF) α by blocking caspase-8 activation (326) by induction of BCL2 (325, 326) and by transactivation of TNF receptor-associated factor 1 (TRAF1), TNF receptor-associated factor 2 (TRAF2), and the inhibitor-of-apoptosis (IAP) proteins c-IAP1 and c-IAP2. It is also involved in autophagy, angiogenesis, metastasis formation, tumor progression, and oncogenesis (121, 246), and is considered a molecular target for cancer therapy (322). It participates in the antioxidant defenses by promoting SOD expression (139). Its inactive form binds to the inhibitor I-κB that masks NF-κB nuclear localization signal and sequesters it in the cytoplasm. Upon irradiation, it can be released and transferred to the nucleus (226).

Specifically, TNFα binding to its receptor (TNFR) leads to a conformational change of TNFR that is accompanied by binding of TNFR type 1-associated DEATH domain protein (TRADD) to TNFR death domain. Two proteins, TRAF2 and receptor-interacting protein (RIP), are then recruited and in turn recruit the IκB kinase (IKK) complex, which is made of IKK-α (IKK1), IKK-β (IKK2), and the regulatory subunit IKK-γ/NF-κB essential modulator (NEMO) (Fig. 13). IKK is then activated and phosphorylates IκB before ubiquitination and proteosomal degradation to release NF-κB (121, 124). Among the plethora of targets with NF-κB responsive element sequences, COX-2, iNOS, and also cytokines (TNFα, IL1, IL6, IL33), chemokines (IL8, MCP-1), VEGF, ICAM1, and VCAM1 have been identified as major factors that can modify the microenvironment and trigger inflammation (95). These molecules may also be involved in oncogenesis (246).

##### b. NF-κB and irradiation

NF-κB can be activated by doses as low as 0.1 Gy of X-rays, but participates in the RIAR (84) also at higher doses (2–50 Gy) (32). RIAR was demonstrated by the observation that a priming dose (generally >0.005 Gy) reduces the detrimental effects of the challenging dose administered a few hours later (186, 333). This can be a beneficial effect for low-dose radiation, but might induce radioresistance during radiotherapy. Indeed, RIAR has been associated with reduced chromosome aberrations, MN formation, and mutation induction (223, 344, 356). RIAR shares similarities with inflammation. It was shown that low irradiation doses lead to ATM phosphorylation that contributes to NF-κB activation and to cell survival, in a process involving also ERK (but not p38/JNK) (4, 151). ATM participates in NF-κB activation by phosphorylating IκB and NEMO (165) that mediate NF-κB inhibition. NF-κB activation by low-dose irradiation leads to the expression of MnSOD 15 min postirradiation, and small interfering RNAs (siRNAs) against MnSOD reduce RIAR (84).

RIAR is also observed in bystander cells. Indeed, a priming dose of 0.02 Gy of γ−rays, delivered 6 h before single-cell microbeam irradiation, inhibited 50% of the bystander effects observed in control cells (no priming dose) (265).

Optimal NF-κB activation is observed at doses between 7 and 10 Gy and with high LET (90–230 keV/μm). In these conditions, NF-κB activation is mediated by ATM recruited at DNA DSB sites (336) and is an alternative pathway leading to p53-mediated signaling. p53 participates in cellular redox status by transactivating p53-induced genes (PIGs) that encode antioxidant molecules (GPx). In irradiated cells, p53 transactivates genes encoding ROS-generating enzymes, such as quinone oxidoreductase (NQO1, PIG3) and proline oxidase (POX, PIG6), BAX, PUMA, and p66SHC, thereby leading to oxidative stress and apoptosis (168, 240). However, the ATM-activated p53 signaling pathway is not required for the bystander response (95), as indicated by the finding that bystander effects can be observed in p53-null cells exposed to gamma rays (350) or to Auger-emitting radionuclides (227).

Several studies reported that common p53-regulated radiation response genes, such as cyclin-dependent kinase inhibitor 1 (*CDKN1A* known as p21^Waf1^), are upregulated in alpha particle-irradiated normal human lung fibroblasts, and also in bystander cells (8, 90). Genes regulated by NF-κB, such as COX-2, IL8, and BCL2A1, also are similarly expressed in bystander and irradiated cells (95).

**FIG. 14.**
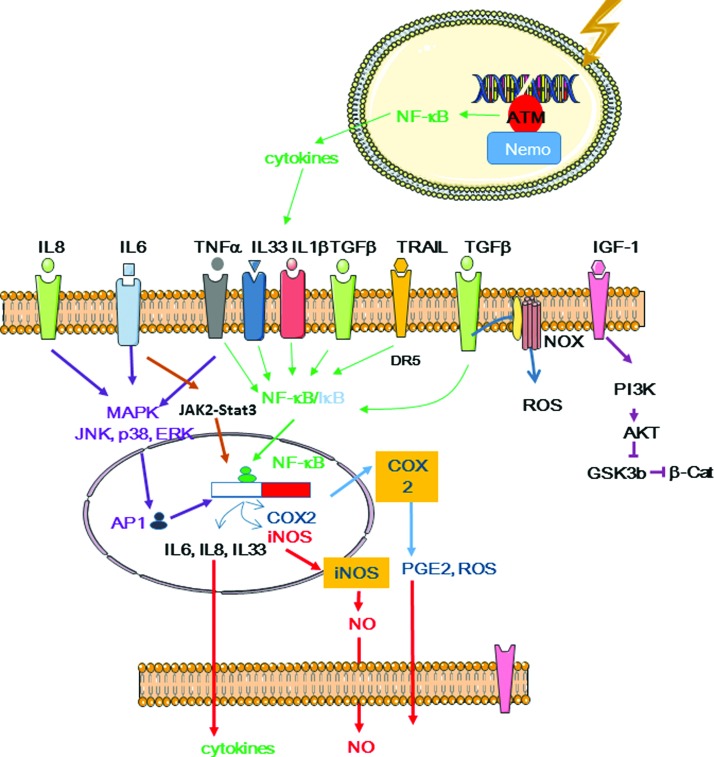
**The NF-κB and cytokine receptor pathways in the bystander response**. DNA DSBs in irradiated cells activate ATM and NEMO that in turn assemble IKK before IκB ubiquitination and proteosomal degradation. Released NF-κB enters the nucleus and induces the transcription of target genes, such as those encoding cytokines. Secreted cytokines can in turn bind to receptors on bystander cells. On binding, receptors will activate NF-κB-responsive element-containing molecules (*e.g.*, cytokines, COX-2, and iNOS), thus contributing to ROS and RNS production and transmission of bystander signals in a self-sustained process. The MAPK and JAK2-STAT3 pathways are also activated by IL8 and IL6, respectively. The TGFβ receptor also can activate NOS. β-Cat, β-catenin; JAK2, Janus kinase 2; PGE2, prostaglandin E2; STAT3, signal transducer and activator of transcription 3; TGF, transforming growth factor. To see this illustration in color, the reader is referred to the web version of this article at www.liebertpub.com/ars

Following gamma or alpha irradiation, activation of NF-κB signaling leads to the production of cytokines and chemokines, such as IL-1α and β, IL-6, TNFα, CXCL1, CXCL2, and CXCL8 (121, 158) (Fig. 14). Activation can occur within few hours following irradiation and might be ATM dependent. A second activation wave can be observed after 24 h and this might be related to receptor binding by secreted cytokines, such as TNFα (24). Therefore, NF-κB could modulate the response of directly irradiated cells and also alert neighboring nonirradiated cells *via* autocrine and paracrine pathways.

NF-κB regulates cytokine production in irradiated cells, but it is controlled by cytokine signaling in bystander cells (151). NF-κB-dependent expression of IL6 *via* IL6-receptor complex activates the Janus kinase 2 (JAK2)-signal transducer and activator of transcription 3 (STAT3) pathway and STAT3-dependent gene expression, thus establishing a link between ATM, NF-κB, and STAT3. STAT3 is a transcription factor located in the cytoplasm and transferred to nucleus on activation. It also maintains constitutive NF-κB activation by prolonging NF-κB nuclear retention (105, 106). NF-κB also transactivates IGF-1 receptor, thereby promoting PI3K/AKT-GSK3b-β-catenin signaling pathways in both irradiated and bystander cells (94, 119, 210) (Fig. 14).

NF-κB can also interfere with the main MAPK signaling. More specifically, it suppresses the JNK cascade and ROS activity (36, 232). It must be noted that TRADD, a TNFR1-associated signal transducer, can also bind to TRAF2 that activates NF-κB and Fas-associated protein with death domain (FADD), two proapoptotic factors, whereas NF-κB is a potent antiapoptotic agent (129).

NF-κB also controls IGF-1 receptor that can activate the downstream (PI3K)-AKT survival pathway in both directly irradiated and bystander fibroblasts (94). In bystander cells, GSK3β phosphorylation by AKT is accompanied by stabilization of beta catenin that acts as a nuclear activator of transcription after GSK3b phosphorylation by WNT signaling (86).

#### 5. The COX-2 and iNOS

The NOS and COX systems include constitutive forms (NOS1, NOS3, and COX-1), which are expressed in many cell types and are mostly involved in housekeeping tasks, and inducible forms (iNOS known as NOS2 and COX-2), which are activated in stress conditions (294). For example, activated macrophages produce clastogenic factors, *via* superoxide and NO, and can induce gene mutations, DNA base modifications, DNA strand breaks, and cytogenetic damage in neighboring cells.

NOS1 and NOS3 are Ca^2+^/calmodulin dependent, while NOS2 is much less. Inducible forms lead to NO production from l-arginine and can be activated by irradiation (181). For example, iNOS activation has been observed as early as 3 h after X-ray irradiation and lasts over 24 h (187). Similarly, NOS1 can be activated by therapeutic doses (2 Gy) and blocks the cytoprotective effects of radiation-induced ERK1/2 activity (161).

COX-2, also known as prostaglandin endoperoxide synthase 2 (PTGS2), converts arachidonic acid into prostaglandin endoperoxide H2 (PGH2), a precursor of PGE2 involved in inflammation. During this reaction, singlet oxygen can also be released. COX-2 is an inducible enzyme responsible for the generation of ROS and of proinflammatory PGE2 (355).

Expression of iNOS (12) and COX-2 (133) is also controlled by NF-κB and is involved in secondary proinflammatory waves following irradiation. Conversely, cNOS can stimulate the early signaling effects of low-dose irradiation (161).

COX-2 is also upregulated in bystander normal human fibroblasts and its inhibition by NS-398 in bystander cells reduces mutagenesis and genetic instability (354). COX-2 is a downstream target of MAPK pathways, such as ERK, JNK, and p38 kinase. Zhou *et al.* showed that inhibition of ERK phosphorylation suppresses COX-2-mediated bystander response (354). Therefore, as a downstream target of the NF-κB and MAPK/AP1 pathways, COX-2 can be induced by a variety of molecules (TNFα, TGFβ, IL6, IL8, IL33, TRAIL, and IGF-1) (293). Finally, radiation-induced activation of NF-κB and of its downstream regulated genes that encode cytokines, such as IL-8, IL-6, COX-2-generated PGE2, and IL-33, can lead to COX-2 activation in bystander cells, *via* autocrine/paracrine stimulation of the NF-κB and MAPK pathways (134).

Zhou *et al.* also showed that radiation-induced bystander effects require mitochondria-dependent NF-κB/iNOS/NO and NF-κB/COX-2/PGE2 signaling pathways (355). While COX-2 leads to ROS production, it is interesting to note that MnSOD is also activated by NF-κB (139), thus providing an antioxidant role for NF-κB in agreement with its antiapoptotic and proliferation properties.

We mentioned above that post-translational modifications of p53, through phosphorylation by ATM and also by other kinases, such as CHK2 and homeodomain-interacting protein kinase 2 (HIPK2), contribute to its stabilization and subsequent induction of transcription of downstream genes involved in cell cycle arrest, programmed cell death, or cell metabolism. Accumulation of p53 also attenuates iNOS induction because p53 interacts with TATA binding protein and/or NF-κB that are essential for *iNOS* expression (85).

### B. Distant/systemic effects

#### 1. Immune response as the mediator of radiation-induced systemic effects

As discussed above, ionizing radiation can cause a variety of effects in cells directly exposed to radiation and also in neighboring and distant nonirradiated cells (*i.e.*, off-target effects or systemic effects). Recent reviews on the topic describe extensively the biological and chemical molecules that induce and/or participate in the propagation of these various off-target effects. The growing number of data supports the necessity of using different approaches (*i.e.*, bioinformatics and meta-analyses) to have a correct picture of the governing mechanisms and type of biological molecules that contribute to the different off-target effects. The most recent bioinformatic studies and meta-analyses have confirmed the interactions between mediators of systemic effects and DNA damage response/repair (DDR/R) components, as well as interactions between pivotal components of the innate immune response, such as pattern recognition receptors (PRRs), and DNA repair proteins (BRCA1, XRCC1, DNA-PK, Ku70/80, and others) (213).

**FIG. 15.**
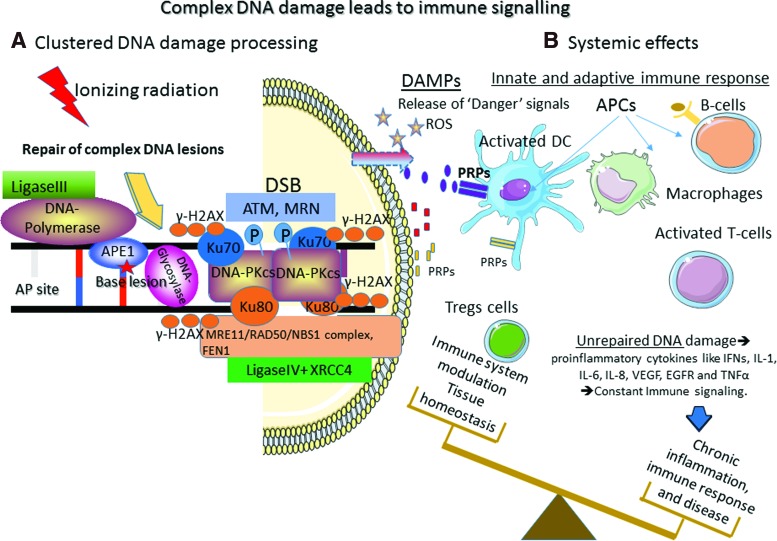
**Complex DNA damage leads to immune signaling. (A)** Repair of a clustered damaged DNA site: a challenging task. On induction by ionizing radiation of complex DNA damage, such as a DSB and two oxidative DNA lesions (a damaged base, *red star*, and an apurinic/apyrimidinic, AP, site), at least two DNA repair pathways and several DNA repair proteins will be activated. For the base damage, BER is the main repair pathway, while for the DSB, only NHEJ will be considered here for simplicity. In all cases, the most basic proteins and enzymes are described. For short-patch BER, a DNA glycosylase will recognize and remove the damaged base and the repair should be completed by the concerted activity of AP endonuclease 1 (APE1), a DNA polymerase, and ligase III to seal the broken ends. In the nearby DSB area (a few bp apart), the Ku heterodimer (Ku70/80) initiates NHEJ by binding to the free DNA ends and engaging other NHEJ factors, such as DNA-PK, XRCC4, and DNA ligase IV, to the DSB site. DNA-PK becomes activated on DNA binding and then phosphorylates a number of substrates, including p53, Ku, and the DNA ligase IV cofactor XRCC4. Phosphorylation of these factors is believed to further facilitate DSB processing. For ligation, the ends must be partially processed by the nucleases Artemis, MRE11/RAD50/NBS1 complex, and FEN-1. Moreover, as shown by advanced fluorescence microscopy, the formation of each DSB is rapidly accompanied by phosphorylation of thousands of histone H2AX molecules (γH2AX). The MRN complex functions as a sensor of DNA ends and activates the ATM kinase that phosphorylates CHK2, p53, and H2AX in flanking chromosomal regions. **(B)**. Systemic effects. Processing of clustered DNA damage can lead to unrepaired and persistent DNA damage that can cause cell senescence or cell death (*i.e.*, apoptosis). This can trigger the extracellular release of different “danger” signals or damage-associated molecular patterns (DAMPs: ATP, short DNAs/RNAs, ROS, heat shock proteins [HSPs], high-mobility group box [HMGB]-1, S100 proteins, and others). DAMPs activate different PRRs, such as TLRs and the formation of inflammasomes, a process that leads usually to inflammation and immune-related pathologies. Interestingly, recent evidence (see section IV.B.1) suggests a direct interaction between different PRRs and DNA repair proteins. Cell damage or death can also lead to the release of several cytokines and chemokines that can regulate immune responses. PRR activation usually results in NF-κB-mediated release of various proinflammatory cytokines, such as IFNs, IL-1, IL-6, IL-8, VEGF, EGFR, and TNFα. The activation of APCs, for instance, dendritic cells and macrophages, will induce primarily the innate immune response (activation of T cells) and most rarely the adaptive immune response (mediated by B cells). In all cases, the constant triggering of the immune system might generate many detrimental systemic effects for the organism. Positive immunomodulation is usually mediated by the action of regulatory (suppressor) T cells (*i.e.*, Treg cells), suppressor macrophages, and immunosuppressive cytokines to maintain overall tissue homeostasis. APCs, antigen-presenting cells; BER, base excision repair; DNA-PK, DNA-dependent protein kinase; EGFR, epidermal growth factor receptor; PRRs, pattern recognition receptors; Treg cells, regulatory T cells; TLRs, Toll-like receptors. To see this illustration in color, the reader is referred to the web version of this article at www.liebertpub.com/ars

Nikitaki *et al.* generated a detailed list of proteins involved in different categories of radiation-induced systemic effects, including the clinically relevant abscopal phenomenon, by using improved methodologies of literature text-mining and various bioinformatic tools. Many of these proteins belong to the DDR complex network and have been found as central hubs in the various protein/protein interaction (PPi) networks, indicating that the key pathways involved in off-target effects are apoptosis, TLR-like, and NOD-like receptor signaling pathways (213) (Fig. 15). PRRs are expressed by the cells of the innate immune system to recognize two classes of molecules and various “danger” signals: pathogen-associated molecular patterns (PAMPs), which are associated with microbial pathogens, and DAMPs, which are associated with cellular components that are produced after cell damage or death. In general, PRRs can recognize abnormal molecular complexes as a consequence of infection, inflammation, or other types of cellular stress (195). DAMPs seem to play an important role in the communication of this stress system-wide and in different organisms, from plants, fish, rats to humans, as reviewed in Ref. (188). Several studies have shown that radiation exposure results in the initiation of various triggering mechanisms associated with the inflammation and immune responses of the host organism, including the release of proinflammatory cytokines and chemokines by monocytes and macrophages. Radiation-induced cytokine gene upregulation is frequently observed, and several genes encoding inflammation-related cytokines (*e.g.*, IFNs, IL-1, IL-6, IL-8, VEGF, EGFR, and TNFα) are considered to be “early response” genes that are activated within minutes to hours after irradiation. This process is considered a genuine “danger” signal in response to radiation injury and it is the main source of *de novo* ROS production. This secondary ROS production can partially explain the late increase in the expression of inflammatory cytokines in irradiated cells (268). Radiation-generated cytokines are responsible for the formation of inflammatory lesions and work together with DAMPs to create the proinflammatory, pro-oxidant microenvironment necessary to characterize this site as a “stress” site and to induce a systemic response. This is expected to promote maturation of dendritic cells and, in cancer treatment, the development of an effector T cell response (*i.e.*, innate response to tumor-associated antigens) (Fig. 15). This process is completed by the attraction of cellular components of the immune system, such as neutrophils, and then macrophages and lymphocytes (268).

Overall, the immune response to radiation is highly related also to the radiation sensitivity (*i.e.*, propensity to undergo apoptosis) of the various immune cell subsets, depending on the lineage, maturity, and activation status. B cells and naive T helper (Th) cells are radiation sensitive, whereas T memory cells, natural killer T cells, and regulatory T cells (Tregs) are more resistant (267). As presented in Figure 15, the final balance between controlled immune response and excessive, pathologically chronic immunogenic response is not easily understood. Certainly, the induction of repair-resistant complex DNA damage on exposure to ionizing radiation is expected to trigger a persistent immune signaling through the continuous activation of the various DNA damage checkpoints that freeze cell cycle until damage is repaired, or direct the cell toward senescence or apoptosis (92). For example, in mice in which the ERCC1-XPF DNA repair endonuclease has been knocked down in all cells or only in adipocytes, DNA damage signaling activates a chronic autoinflammatory response resulting in fat depletion. This response is cell autonomous and most importantly requires ATM, a well-known DNA damage signaling molecule (146).

The role of the innate immune system in coordinating these response types is a rapidly emerging research area (146). As discussed by Gasser *et al.* (93), the immune system uses mislocalized or damaged DNA to discover infected or otherwise diseased and malfunctioning cells. Conversely, genomic DNA and mtDNA in healthy cells are usually overlooked by the DNA recognition pathways. Furthermore, cells have a limited ability to degrade mislocalized DNA and to repair any damaged DNA, possibly to prevent the unplanned activation of DNA sensor pathways and of the inflammation and immune systems. It is generally accepted that there is a multivariable alliance between DDR and immune signaling and the modulating parameters are not always understood (234). One of the most coherent immune rebalancing activities involves shifting the T cell equilibrium toward regulatory (suppressor) T cells (*i.e.*, Treg cells), and this can occur after radiotherapy (267). In addition, immunomodulation can be achieved also by the action of suppressor macrophages and immunosuppressive cytokines to secure overall tissue homeostasis (268).

#### 2. Abscopal effects: historical changes and clinical evidence

Although modern radiotherapy is getting progressively closer to its ideal target size or volume, still there are unresolved questions; particularly, it is not clear whether radiation effects are be really limited and localized. The idea of distant, abscopal effects was introduced for the first time by Mole in 1953 (196). Abscopal is made of the “*Ab*” prefix, which means “away from,” and “scopos” (Latin), which means shooting mark or target. Therefore, this adjective describes the idea of “off-target” or “away from target” effects and supports the hypothesis of the interdependence of all body cells (196). As suggested by Mole, this practically means that damage to one cell will unavoidably affect the body as a whole. This initial hypothesis was based on the finding that in rats, the thyroid gland synthetic activity was reduced to 25% of the normal 3 days after localized irradiation with 6–10 Gy. However, this was not caused by whole body irradiation or direct irradiation of the thyroid or the pituitary gland. Moreover, the thyroid function reduction was observed only when a sufficiently large volume of the abdomen was irradiated (196).

Although the idea of abscopal effects seems logical, it has been disregarded in radiotherapy for many years. It took about 50 years to show clear mechanistic evidence, at least in mice, about the immune system's role in this phenomenon (72). Specifically, mice bearing one syngeneic mammary carcinoma (mouse 67NR cells derived from mammary gland malignant neoplasms) in each flank were treated with the growth factor Flt3-Ligand (Flt3-L; daily for 10 days) to boost dendritic cell production, after local radiotherapy (or not) to only one of the two tumors (single dose of 2 or 6 Gy). Growth of the nonirradiated tumor was impaired only in mice that received unilateral radiotherapy and Flt3-L, but not in animals treated only with Flt3-L. This result indicates that immunity is involved in the abscopal effect. Radiotherapy immunostimulatory effects have created a wide interest due to the preclinical and clinical observations that tumor-localized radiotherapy can occasionally prompt anticancer immune responses that facilitate the regression of distant and nonirradiated metastatic tumors, as recently reviewed in Wennerberg *et al.* (332).

Although circumstantial evidence existed since the 1950s, abscopal effects are still considered in some cases as unexplained, obscure, or of limited clinical value due to the high specificity and dependence on the type of host organism, tumor, radiation, and so on. In the 1960s, Law and Mole (159) showed direct (*i.e.*, targeted) and abscopal effects of X-ray radiation on the thymus of weanling rats. More recently, many studies using primarily mice or rats described a variety of “off-target” effects (34, 57, 137, 155, 156, 184, 216, 320, 321). Additional work is needed to precisely evaluate the type and degree of abscopal effects in a variety of model organisms [mice (264), rats, earthworms (197)] and different targeted organs (head, thorax, *etc*.) (101, 152, 190, 236). Very recently, Ventura *et al.* exploited synchrotron radiation to study targeted radiation-induced “off-target” effects in C57BL/6 mice (317). Under different radiation settings, irradiation of a small leg area induced pronounced persistent systemic genotoxic effects (such as complex DNA damage and cell apoptosis) also in nonirradiated areas. These genotoxic events were accompanied by changes in the plasma concentrations of macrophage-derived cytokines, eotaxin, IL10, TIMP1, VEGF, TGFβ1, and TGFβ2, as well as changes in the tissue proportion of macrophages, neutrophils, and T lymphocytes, underlying the strong links between response to radiation and immune system.

It is now quite safe to suggest that local irradiation of a tumor leads to cell death and tissue damage and the release of ROS, cytokines, and danger signals, several of which can trigger an innate immune response (144), although clinical evidence is still rare. One of the first clinical evidences was the description in 2009 of an abscopal effect in a patient with chronic lymphocytic leukemia during radiation therapy (157). One week after X-ray radiotherapy, the lymph nodes in the neck that were not irradiated and distant from the irradiated area started to regress, and after 2 weeks of radiotherapy, they showed complete regression (157). The same year, another patient was diagnosed with acute radiation pneumonitis and other signs of radiation toxicity after spine irradiation. The authors suggested that this should alert clinicians on the toxicity risk for nontarget organs that receive low-dose or zero radiation (283). For a list of the reported clinical cases concerning abscopal effects in patients with nonhematological malignancies and treated by conventional radiation (patient characteristics, treatment strategy, and outcomes), the reader can refer to a relatively recent review by Siva *et al.* (291).

Currently, the idea that abscopal effects exist and can be modulated primarily *via* the immune system is progressively more accepted (87, 125, 174, 261). Encouraging results based on case reports and preclinical data suggest that radiotherapy and immunotherapy may synergize to create a scenario of off-target responses away from the radiation field that could be beneficial for the patient (2, 51, 60, 70, 71, 116, 171, 273, 301). DDR mechanisms and inflammatory responses have been recently observed in patients undergoing radiotherapy for nonsmall-cell lung cancer. The observed abscopal effect was also linked to changes in the plasma levels of MDC/CCL22 and MIP-1alpha/CCL3 cytokines (290). Undoubtedly, one of the major breakthroughs is the finding that the combination of radiation and immunotherapy to boost the immune system (*e.g.*, with the human monoclonal anti-CTLA-4 antibody ipilimumab) induces immune-mediated abscopal effects in poorly immunogenic preclinical tumor models and in patients with metastatic melanoma or other cancer types (62, 100, 237). The suggested mechanism for this therapeutic type is that the anti-CTLA4 monoclonal antibody binds to CTLA4 expressed on the surface of T cells and inhibits the CTLA4-mediated downregulation of T cell activation (75). This consequently leads to a boosted cytotoxic T lymphocyte (CTL)-mediated synergistic immune response against cancer cells and high tumor immunity, especially when combined with radiation. Currently, the National Cancer Institute (NCI) lists only one Phase I clinical trial for solid cancers or lymphoma. This is a multicenter study to evaluate the safety of an anti-CTLA-4 human monoclonal antibody (AGEN1884) and to estimate the maximum tolerated dose in subjects with advanced or refractory cancer (Clinicaltrials.gov ID: NCT02694822).

Another early example of regression of hepatocellular carcinoma was observed after radiotherapy (total dose of 36 Gy) for bone metastasis (220). Doses of 2 and 6 Gy have been used in combination with the dendritic cell growth factor Fms-related tyrosine kinase 3 (FLT3) or with injection of dendritic cells in tumors as immune therapy (72, 150). In mice with melanoma tumors, a single fraction of 15 Gy showed similar results as 3 × 5 Gy and was accompanied by the generation of tumor antigen-specific effector cells that traffic to the tumor (175). The combination of anti-CTLA4 antibodies with fractionated irradiation (3 × 8 Gy or 5 × 6 Gy), but not single-dose irradiation (20 Gy), showed tumor growth delay outside the field of irradiation in preclinical models (300).

### C. Off-target effects: an integrated cell response to radiation: conclusion

It was initially thought that the harmful effects of ionizing radiation were caused by clusters of DNA lesions, involving DNA DSBs, in irradiated cells. However, several observations progressively modified this DNA-centered paradigm view of radiobiology. First, the yields of radiation-induced ROS and RNS formation and subsequently of DNA lesions were relatively low compared with the endogenous production [mainly by mitochondria, NAD(P)H oxidases]. Second, DNA lesions involving DDR activation were observed in cells that had not been traversed by radiation, but that were close to irradiated cells (bystander effects). These bystander effects are the consequence of oxidative stress signals initiated in irradiated cells and propagated to neighboring cells. In this context, not only the initial nuclear DNA breaks but also all the cell compartments (cell membrane, mitochondria, ER) and associated complex networks that involve Ca^2+^ release and ROS/RNS production through NF-κB, iNOS, and COX activation have to be considered. Increased oxidative stress in irradiated cells and its transmission also to neighboring cells can amplify the initial response.

Distant systemic effects of radiation, which in the clinic are often called abscopal effects, are generally accepted nowadays and probably complete the picture of radiation effects in the whole body. Similarly to bystander effects, they are initiated by local DNA damage in irradiated cells or tissue. This can be considered the triggering effect that marks the irradiated area as a “stress” area in the body. Distant radiation effects involve the release of short and long distance messengers to convey the stress signal to distant sites by the mediation of well-conserved inflammatory and immune response networks. In the clinic, the most extreme manifestation of this phenomenon is tumor shrinkage in distant sites not reached by irradiation. This knowledge could pave the way to clinical applications of this systemic effect of radiation treatment.

## V. Benefit/Risk Analysis

In the clinic, irradiation is used to destroy tumor cells by triggering cell death mechanisms. Innovative technologies and procedures have been developed in conventional EBRT, and the use of radionuclides for both imaging and therapy has progressively gained interest in the last two decades. Predicting the potential consequences of off-target effects is required for both radiation effectiveness and radiation risk assessment (247).

### A. Target theory

The prediction of therapeutic efficacy and of side effects of radiation exposure relies on the establishment of dose/effect relationships between radiation dose and tissue reactions. Most radiobiological studies have used EBRT, generally with gamma and X-rays (244). The biological effects induced by radiation at medium (0.5–5 Gy) to high doses (5–15 Gy) are quite well known (140). They are usually subdivided as follows: (i) nonstochastic effects (also called deterministic or tissue reaction effects) that occur above a certain threshold (>0.5 Gy) and the severity of which increases with the dose; and (ii) stochastic effects (genetic risks in offspring and cancer) for which there is no threshold, but only a probability of occurrence. The tumor tissue response to radiation (assimilated to the tumor control probability, TCP) belongs to the nonstochastic effects category. In EBRT, it is proportional to the dose according to a sigmoid curve. At the molecular level, this curve reflects the eradication of the most radioresistant clonogenic malignant cells present in the tumor. TCP can be described using mathematical models, and cell killing can be analyzed using the Poisson law as statistical model. The “*N*” in the Poisson law defines the probability for targets to be hit *N* times (160, 351). Therefore, the survival fraction (S) is the survival probability [*p*(0)] for a cell that receives no lethal hit [S = *p*(0) = exp(−*Ñ*), *Ñ* being the average number of lethal lesions] (324).

This model has been later replaced by a linear quadratic (LQ) model [S = exp(−αD − βD^2^) where α and β are constant parameters and D is the dose. *Ñ* contains linear (αD) and quadratic (βD^2^) terms, indicating that lethal lesions could be caused by one single track (αD term) or two tracks (βD^2^ term). In this model, two tracks (βD^2^ term) produce less severe lesions (called sublethal lesions) that, taken individually, could be repaired in the absence of the second track deleterious effects. Conversely, the single track of the (αD) term corresponds to a lethal lesion. The (α/β) ratio refers to the cell repair capacity and radiosensitivity. This formulation has the advantage of considering both physical hits and molecular response (148). The α/β ratio has been determined clinically for normal tissues and tumors (17, 18, 63, 64, 138, 288). Tissues with small α/β ratio values have greater repair capacity and are more sensitive to dose fractionation than tissues with high ratio values.

In this context, tumor eradication means that no clonogenic cell survives. Therefore, TCP is strictly proportional to the number of clonogenic cells, depending on the dose: TCP = exp(−*N*s) where *N*s is the number of clonogenic cells and contains the (−αD − βD^2^) expression (138).

### B. The clinical relevance of off-target effects might be radiotherapy dependent

On the contrary, a general rule about the benefit/risk ratio of off-target effects in radiotherapy might not exist because this response is influenced by many parameters: type of radiotherapy, dose ranges, dose rates, dose fractionation, and radiation type (photons *vs.* charged particles). In conventional EBRT, X-rays (usually 2 Gy fractions for 1–2 min) are delivered over several days and weeks to reach the final tumor biological effective dose. Improvement in conforming irradiation to the tumor volume to protect healthy tissues has led to the development of 3D-conformal radiation therapy and more recently to intensity-modulated radiation therapy (IMRT) and image-guided radiation therapy using computed tomography, positron emission tomography, or magnetic resonance imaging. Other irradiation modalities, including tomotherapy, stereotactic radiosurgery, stereotactic body radiation therapy (SBRT), involved-field radiation therapy, have been developed (247). Therefore, it is becoming difficult to extrapolate radiobiology notions for conventional treatment with 2 Gy·min^−1^ to other irradiation methods, for instance, SBRT with high doses of radiation. High LET particles, such as heavy carbon ions and protons, are now routinely used in the clinic (21, 222). They take advantage of the spread out Bragg peak (145).

Radionuclide therapy is another procedure where off-target effects have to be considered. Radionuclides are used for the treatment of various diseases, such as thyroid cancer with ^131^I, lymphoma with radiolabeled anti-CD20 monoclonal antibodies (^90^Y-ibributomab tiuxetan; Zevalin^™^), neuroendocrine tumors with *^177^Lu*-DOTA0-Tyr3-octreotate (Lutathera^™^), neuroblastoma, carcinoid tumor, pheochromocytoma, paraganglioma with ^131^I-meta-iodobenzylguanidine (MIBG), bone metastases from prostate cancer with ^223^RaCl_2_, and hepatocellular carcinoma with ^90^Y microspheres. Other radionuclides are used for palliative treatment of bone metastases (^89^Sr, ^153^mSr, ^186^Re, ^188^Re, ^223^Ra) (33), and many other radiolabeled antibodies or peptides are currently assessed in clinical trials.

The particularity of radionuclide therapy is that radionuclides are targeted by conjugation with peptides or antibodies and are then injected in the circulation. The dose is delivered over several hours and days, at a relatively low-dose rate. The delivered dose can be strongly heterogeneous because of tumor-site accessibility issues and also because of the particles used (beta, alpha, and Auger electrons). Heterogeneity also occurs at the whole-body scale because the whole body may be irradiated (at variable doses), although the highest dose is delivered to the tumor. Moreover, the interplay between the radiation effects and the biological effects of the vector should also be considered (243). Because of its physical features, radionuclide therapy could be, to some extent, assimilated to low-dose rate brachytherapy using sealed sources of ^125^I for prostate cancer treatment. Moreover, as some of the radionuclides (*e.g.*, ^223^Ra, ^213^Bi, ^225^Ac, ^211^At, ^212^Pb/^212^Bi) are high-LET particle emitters, some forms of targeted radionuclide therapy show similarities, to some extent, with external radiotherapy using other types of high-LET particles, such as heavy ions or protons. Therefore, there is not one form of radiotherapy but many. Consequently, the assessment of the contribution of off-target effects should be considered a major field of research without a universal rule. For instance, Mairs's team showed that the bystander response to the same MIBG molecule labeled with alpha, Auger, or beta emitters produces different effects (31).

### C. Bystander effects and radiation protection

There is evidence of *in vivo* (16, 33, 228, 243, 339) and *ex vivo* (16) bystander effects during EBRT and radionuclide therapy. In conventional EBRT, bystander effects are mainly expected to contribute significantly to the cell outcome at low doses (<0.5 Gy) (248, 274). This is due to the observation that targeted effects increase with the dose, while bystander effects saturate as the dose increases. Therefore, targeted effects predominate at high dose compared with bystander effects. Conversely, Mothersill and Seymour showed that *in vitro*, cell death caused by gamma-ray doses comprised between 0.01 and 0.5 Gy is only due to bystander effects (274). However, bystander effects are also observed after high-dose irradiation (26).

At doses below 0.1–0.2 Gy, only stochastic effects, including cancer, are relevant for assessing the tissue response. Such doses can be encountered during environmental exposures and also during treatment in nontumor tissues located at the margin of the irradiation field, or in the presence of dose gradients, particularly in IMRT, tomotherapy, and heavy ion particle therapy. It also concerns diagnostic radiological procedures and possibly also nuclear medicine. It must be also noted that the assessment of the dose delivered to the tumor and healthy tissues is more difficult and challenging during radionuclide therapy than EBRT, making risk assessment even more complicated.

The effects of such low doses, essentially cancer induction, could be predicted by extrapolating from assessments at high dose and by correcting for factors that account for low-dose rate and low dose, according to a linear no-threshold (LNT) model. The LNT model assumes that detrimental effects decrease according to the dose rate and are proportional to the dose. According to the “target theory” that relies mainly on a DNA-centered view of radiobiology (3), detrimental effects of radiation (mutagenesis, carcinogenesis, and cell death) are only observed in irradiated cells (more precisely in their nucleus) and in their progeny. This view is now challenged by the identification of harmful effects of radiation also in nonirradiated cells (off-target effects) and their amplification. However, the subject is still a matter of debate because there are many experimental and epidemiological evidences showing that it makes sense to extrapolate the risk from high to low doses. Nevertheless, many other studies highlight some of the weaknesses of the target theory (140).

The theory behind off-target and bystander effects considers that cells can die without being crossed by particles, that is, without having absorbed any energy from radiation. The ultimate consequence is that irradiated cells cannot be considered anymore as an independent entity, the final fate of which depends solely on the delivered dose and on their intrinsic radiosensitivity, which is one of the bases of the “target theory.” Conversely, the interplays with the microenvironment and even with the whole body through systemic communication that involves the blood and lymphoid compartments have to be considered.

### D. The biology of low dose and low-dose rate might differ from that of high dose and high-dose rate

Besides bystander effects, several other phenomena, such as inverse dose rate effect and low-dose hypersensitivity, challenge the LNT model and risk assessment. For example, genomic instability frequency is higher at low than at high doses (309), and bystander effects have also been observed at low EBRT doses and after one single alpha particle track. As mentioned above, the RIAR shows that a priming dose (generally >0.005 Gy) reduces the detrimental effects of the challenging dose administered a few hours later (140, 275).

Therefore, it is likely that extrapolating data from high to low dose cannot be corrected solely by considering that cells have more time to repair DNA DSBs at low-dose rate, but by considering to some extent different biological mechanisms.

### E. Off-target effects and radiotherapy efficacy

The existence of off-target effects might be an advantage for tumor eradication, although they are still difficult to control. One of the challenges is now to define the best radiotherapy plan (dose, dose rate, LET) and combination for promoting these effects. There have been several approaches (247) based on gene therapy to introduce NOS2 (328) and to use radiation-iNOS promoters (334). Gene therapy with human telomerase promoters associated with targeted radiotherapy using ^131^I-MIBG radionuclides has also been investigated by Boyd *et al.* (31). Some strategies focused on DNA because DNA repair in bystander cells is different compared with irradiated cells and involves ATR upstream of ATM (38, 96), highlighting the role of stalled DNA replication forks. In this respect, BRCA1, FANCD2, and CHK1 have been identified as potential molecular targets in bystander cells (37).

The parameters affected by new clinical practices are dose and fractionation (hypo- and hyperfractionation). In some way, radionuclide therapy could be assimilated to hyperfractionated therapy. It is clear that dose and dose fractionation can modify the contribution of off-target effects to the final outcome. It was shown that the bystander response resulting from fractionated irradiation is different in tumor and healthy tissues (203).

The dose influence on the bystander response was reviewed by Tomita and Maeda (307). Bystander cell death induced by doses as low as 0.01 Gy of low LET radiation (γ-rays from ^60^Co) is similar to the cell death caused by exposure of nonirradiated cells to conditioned medium from cells irradiated at the same dose. It seems that there is a threshold above 2–3 mGy and bystander response reaches a plateau at 0.3 Gy (169). The influence of dose and dose rate on abscopal effects was reviewed by Rodel *et al.* (258). Radiation-induced immunosuppressive effects are observed at relatively low and clinically relevant doses (<0.5–1 Gy) and include depletion of immune cells, polarization of M2 macrophages, and increase in the number of radioresistant Treg cells. Irradiation immunosuppressive effects have been used as early as 1898 to treat chronic inflammatory diseases (X-ray-treated polyarthritis). Currently, radionuclides (^89^SrCl_2_, ^153^Sm-EDTMP, or ^186/188^Re–HEDP) are proposed for the palliative treatment of bone metastases. Indeed, the palliative effects of low doses should be mediated by innate immune cells, such as macrophages. In function of their microenvironment, macrophages can behave as proinflammatory actors (classically, the activated M1 phenotype) that secrete proinflammatory cytokines (IL-6, TNFα, IL-1), and chemotactic factors (IL-8 and CCL20), or as anti-inflammatory cells (M2 macrophages). Moreover, both initiation and resolution of inflammation participate in tissue homeostasis. In this way, macrophages are involved in several functions, including phagocytosis (CD31), antigen presentation through CD40 receptors, secretion of cytokines (TNFα or IL-1 under the control of NF-κB), ROS release, and RNS production through iNOS.

At doses of 0.5 and 0.7 Gy, secretion of proinflammatory cytokines (*e.g.*, TNFα) is reduced in macrophages derived from human THP-1 cells and in mouse macrophages stimulated by LPS (313). The TNF family is involved in ROS production required for pathogen killing by phagocytes. In turn, ROS activate NF-κB, leading to additional TNFα production. However, although iNOS expression in macrophages is increased in inflammation, radiation seems to decrease its expression in some cases (268). This could be mediated by an increase in the stress protein heme oxygenase 1 (HO-1) (123). Therefore, the anti-inflammatory effect of low doses of ionizing radiation could be mediated by reduction in NO production. Moreover, low-dose irradiation can promote secretion of the anti-inflammatory cytokines TGFβ1-3 by M2 macrophages (258, 312). Production of IL-1β also is decreased in macrophages of radiosensitive Balb/c mice exposed to 0.5 or 0.7 Gy of ionizing irradiation (X-ray) (88). The authors mentioned that 0.5 Gy gamma radiation causes upregulation of MAPK phosphatase-1 (MKP-1), leading to inactivation of the p38 MAPK and suppression of TNFα production.

Conversely and as previously discussed, abscopal effects have been described in preclinical models and in patients at higher doses than those causing bystander effects (2–40 Gy) (258).Their occurrence and contribution to the final antitumor efficacy depend on the tumor and its microenvironment. Moreover, they are more pronounced when using therapeutic combinations that activate the immune system, such as injection of dendritic cells (87, 261). Currently, the critical factors orchestrating the overall response of the tumor and of the organism to irradiation are not clearly defined. Nevertheless, it is accepted that there is a synergy between tumor response, radiation toxicity, and immune system.

### F. Benefit/risk analysis: conclusion

The off-target effects of radiation are a major concern both for radiotherapy and radiation protection. The relative contribution of off-target effects to the final therapeutic efficacy and to the detrimental effects of radiation might depend strongly on the type of exposure (*e.g.*, dose, low *vs.* high LET, protracted *vs.* acute irradiation, homogeneous *vs*. heterogeneous irradiation). Off-target effects are relatively difficult to predict and to control, but they can be advantageous for tumor treatment. In EBRT, bystander effects should be predominant at low dose (0.5 Gy), compared with targeted effects, the contribution of which increases with dose and becomes predominant at high doses. However, the contribution of off-target effects might vary in function of the radiotherapy type (*e.g.*, radionuclide therapy), although the response is expected to reach a plateau. On the contrary, abscopal effects could be exploited at higher doses, by using appropriate treatment combinations and doses.

Radiation protection systems rely on the LNT model. Although it is now clear that off-target effects (*e.g.*, bystander and abscopal effects) must be considered both at low and high doses, it is still not known whether epidemiologically, these effects will be traduced statistically to an increase or decrease of the risk for healthy tissues. Although new findings highlight the possible role of off-target and delayed cellular effects, UNSCEAR considers that the current risk estimates for irradiation-induced cancer and hereditary effects in humans do not need to be changed.

## VI. General Conclusion

In this review, we presented in an analytical way the current status of knowledge in the area of off-target and away-from-target ionizing radiation effects with special emphasis on their role on radiation therapy and clinical applications. After years of intensive *in vitro* and *in vivo* (animals) research and circumstantial clinical evidence in humans, the idea of off-target effects has gained high interest and is increasingly accepted by the scientific community. It is certainly considered a paradigm shift from the target theory in radiation biology where biological important components of the cells must be directly hit from radiation to show a radiation response.

Undoubtedly, radiation harmful effects are linked to the induction of a high percentage of clustered lesions that destroy the target (*i.e.*, any cellular component, but particularly DNA, proteins, and the cell membrane with its lipids). Although in this review, we focused on DNA, we cannot disregard the importance of other cell targets that could contribute to the radiation response and systemic effects in the organism. We also believe that ionizing radiation effects must be evaluated at the whole organism level and not just in the directly hit area. Therefore, we discussed the prevailing mechanisms underlying off-target effects, particularly the release of radical species (ROS/RNS) from the irradiated area, induction of the DDR, and repair and activation of a variety of danger/stress signals that relate to the various forms of radiation injury and apoptosis (Figs. 6 and 14). The result is usually a proinflammatory response and in many cases, the activation of the immune system, a process that shows many similarities with the stress response on damage or pathogen intrusion. We described all the key molecules that participate in this process, such as the NF-κB/COX-2 signaling pathways, and the relevant experimental evidence. We also discussed the emerging view on the role of complex DNA damage because from an evolutionary point of view, this is the only discriminating factor between irradiation-induced and endogenous oxidative stress-induced damage (Fig. 15).

We then discussed the clinical importance of these molecular mechanisms and of the association between targeted and off-target radiation mechanisms. We described the existing evidence for the systemic nature of radiation effects (*i.e.*, abscopal phenomenon). In our opinion, abscopal effects are a genuine manifestation of the holistic nature of the body response to radiation, even when administered to a localized area. Strong *in vivo* evidence in various model organisms (from animals to humans) supports the induction of radiation effects at sites distant from the irradiation site. Many of these studies reported tumor regression and the involvement of the innate immune system, thus underlying the strong links between the radiation response mechanisms (*e.g.*, DNA damage sensing, DNA repair, and apoptosis) and immune system activation. The involvement of the immune system is supported by the finding that the combination of radiation with immune system-boosting drugs, such as the anti-PD-1 (MEDI0680) or anti-CTLA-4 (AGEN1884) monoclonal antibodies, led to a reduction of the size of a metastatic nonirradiated tumor and of cancer spread in the body. These new treatments that combine radiation and immunotherapies are based on radiation biology data showing that the radiation response occurs at the whole-organism level, something which was often disregarded up to few years ago. Although many questions on the propagation mechanisms of off-target effects still exist, these new concepts open the way for the development of more efficient radiation-based immunotherapies and other adjuvant therapies.

## VII. Key Points

Cell damage is mediated by the direct interaction of ionizing radiation with water and cellular constituents, such as DNA, lipids, and proteins.

Radiation generates “danger” signals that propagate from irradiated to nonirradiated cells, leading to off-target (bystander and abscopal/distant) effects.

Redox mechanisms also play a key role in both targeted and off-target radiation effects.

NF-κB is essential for triggering the self-sustained production of reactive oxygen and nitrogen species (ROS and RNS) that are involved in both targeted and off-target radiation effects.

The level of damage produced by radiation is relatively lower than that caused by endogenous oxidative stress.

The harmful effects of radiation are explained not only by the induction of closely spaced (clustered) DNA lesions but also by the amplification of the initial radiation-induced cellular response.

Immune cells can be recruited and contribute to the distant (abscopal) effects of irradiation with potential clinical implications.

Differently from targeted effects (*e.g.*, the induction of DNA damage), off-target effects are not strictly dose related.

Although there is evidence of off-target effects existence *in vivo* through the mediation of the immune system and of their applicability in cancer immunotherapy (*e.g.*, use of immune checkpoint inhibitors), they are still difficult to predict and control.

Off-target effects contribution is likely to depend on the irradiation physical parameters (type and level of damage) as well as on the type of tissue and organism.

Radiation-induced systemic phenomena (as opposed to localized, direct damage) represent a radiobiology paradigm shift and must be taken into account in radiation protection as well as in radiotherapy.

## Abbreviations Used

3D: three dimensional
4-HNE: 4-hydroxy-2-nonenal
53BP1: p53-binding protein 1
8-oxodGuo: 8-oxo-7′8-dihydro-2′-deoxyguanosine
ASMase: acid sphingomyelinase
ATM: ataxia-telangiectasia mutated
ATR: ATM- and RAD3-related
BRCA1: breast cancer type 1
CHK1: checkpoint kinase 1
CHK2: checkpoint kinase 2
cNOS: constitutive nitric oxide synthase
COX-2: cyclooxygenase 2
c-PTIO: 2-4-carboxyphenyl-4,4,5,5-tetramethylimidazoline-1-oxyl-3-oxide
CtIP: C-terminal-binding protein 1-interacting protein
CTL: cytotoxic T lymphocyte
CuZnSOD: Cu–Zn-dependent superoxide dismutase
Cxs: connexins
DAMPS: damage-associated-molecular-patterns
DDR: DNA damage response
DNA-PK: DNA-dependent protein kinase
DSBs: double-strand breaks
dsDNA: double-stranded DNA
DUOX: *dual oxidase*
EBRT: external beam radiotherapy
EGFR: epidermal growth factor receptor
eNOS: endothelial nitric oxide synthase
ER: endoplasmic reticulum
ERK: extracellular signal-regulated kinases
ETC: electron transport chain
FAD: flavin adenine dinucleotide
FANCD2: Fanconi anemia group D2 protein
FAK: focal adhesion kinase
FAT: FRAP-ATM- TRRAP
FATC: FAT C terminal
Flt3-L: Flt3-Ligand
GJIC: gap junction intercellular communication
GPCRs: G-protein-coupled receptors
GPx: glutathione peroxidase
GSH: glutathione
H_2_O_2_: hydrogen peroxide
HOCl: hypochlorous acid
HRR: homologous recombination repair
IAP: inhibitor-of-apoptosis
IGF-1: insulin-like growth factor 1
IKK: IκB kinase
IMRT: intensity-modulated radiation therapy
iNOS: inducible NO synthase
IP_3_: inositol trisphosphate
JNKs: c-Jun N-terminal kinases
JAK2: Janus kinase 2
LET: linear energy transfer
L-NAME: NG-nitro-L-arginine methyl ester
LNT: linear no-threshold
LPS: lipopolysaccharide
MAPK: mitogen-activated protein kinase
MDC1: mediator of DNA damage checkpoint protein 1
MDA: malondialdehyde
MDM2: mouse double minute 2 homologue
MDS: multiple damage sites
MIBG: meta-iodobenzylguanidine
MMP: matrix metalloproteinase
MN: micronuclei
MnSOD: manganese-dependent superoxide dismutase
MRN: MRE11–RAD50–NBS1
mtDNA: mitochondrial DNA
NAD(P)H: nicotine adenine dinucleotide phosphate
NEMO: NF-κB essential modulator
NF-κB: nuclear factor kappa B
NHEJ: nonhomologous end joining
NO: nitric oxide
NOS: nitric oxide synthase
NOX: NAD(P)H oxidase
O_2_^−•^: superoxide anion
ONOO^−^: peroxynitrite
Panxs: pannexins
PGE2: prostaglandin E2
PI3K: phosphatidylinositol-3-kinase
PIG: p53-induced genes
PKC: protein kinase C
PLC: phospholipase C
PP2A: protein phosphatase 2
PRRs: pattern recognition receptors
PUFA: polyunsaturated fatty acids
PUMA: p53-upregulated modulator of apoptosis
RIAR: radiation-induced adaptive response
RNS: reactive nitrogen species
ROS: reactive oxygen species
SBRT: stereotactic body radiation therapy
SOD: superoxide dismutase
SSBs: single-strand breaks
STAT3: signal transducer and activator of transcription 3
TCP: tumor control probability
TGF: transforming growth factor
TLR: Toll-like receptor
TNF: tumor necrosis factor
TRADD: TNFR type 1-associated DEATH domain protein
TRAF2: TNF receptor-associated factor 2
TRAIL: TNF-related apoptosis-inducing ligand
Treg cells: regulatory T cells
UNSCEAR: United Nations Scientific Committee on the Effects of Atomic Radiation
VDACs: voltage-dependent anion channels
